# Multichannel 3D-Printed Bioactive Scaffold Combined with Small Interfering RNA Delivery to Promote Neurological Recovery after Spinal Cord Injury

**DOI:** 10.34133/research.0951

**Published:** 2025-10-21

**Authors:** Jingjia Ye, Fenglu Li, Zhengfa Wen, Junsheng He, Gaoxing Pan, Xinrang Zhai, Linran Song, Xianzhu Zhang, Xuefei Zhou, Xudong Yao, Yanlang Wang, Jin Zhang, Wei Wei

**Affiliations:** ^1^ Department of Orthopedics, Center for Regeneration and Aging Medicine, the Fourth Affiliated Hospital of School of Medicine, and International School of Medicine, International Institutes of Medicine, Zhejiang University, Yiwu, Zhejiang, 322000, P. R. China; ^2^ Qingyuan Innovation Laboratory, 1 Xueyuan Road, Quanzhou 362801, P. R. China; ^3^ College of Chemical Engineering, Fuzhou University,, Fuzhou 350108, P. R. China; ^4^ School of Chemistry and Chemical Engineering, Nanjing University of Science and Technology, Nanjing, Jiangsu, 210094, P. R. China; ^5^ Department of Sports Medicine of the The First Affiliated Hospital, and Liangzhu Laboratory, Zhejiang University School of Medicine, Hangzhou 310003, P. R. China; ^6^ School of Advanced Manufacturing, Fuzhou University,, Jinjiang 362200, P. R. China

## Abstract

Enhancing axonal regeneration holds promise for restoring neural circuits and locomotion function after spinal cord injury (SCI), while precise guidance of micrometer-scale axons to their natural regions remains a critical challenge. To address this problem, we developed an integrated 3D-printed scaffold featuring internal parallel channels infused with a bioactive hydrogel containing laminin-derived chimeric RADA_4_-IKVAV peptide. This scaffold combined physical guidance cues with molecular modulation synergistically by constructing an incorporated small interfering RNA delivery platform targeting phosphatase and tensin homolog. Comprehensive validations via immunohistochemistry, single-cell RNA sequencing, and behavioral assays demonstrated that this approach effectively protected surrounding tissues in lesion area, enhanced ability of axonal regeneration, and improved locomotion functional recovery of rats significantly. Mechanistic investigations further revealed that the introduced IKVAV peptide specifically up-regulated Ephrin/Eph signaling pathway genes, driving establishment of functional axon networks. Overall, this strategy potentially helps to develop new clinical approach for efficiently treating SCI.

## Introduction

Spinal cord injury (SCI) represents one of the most devastating neurological conditions. Limited regenerative capacity of central nervous system (CNS) neurons combined with a pathological injury microenvironment contributes to varying degrees of permanent neurological deficits [1,2]. Although many strategies including stem cell therapy and neurotrophic factor delivery have already targeted extrinsic mechanisms of axonal regeneration, achieving robust regeneration of injured axons still remains challenging [3,4]. Phosphatase and tensin homolog (PTEN) as a tumor suppressor gene constitutively inhibits the mammalian target of rapamycin (mTOR) pathway, which is a critical signaling cascade for regulating cell growth and axonal extension [5]. PTEN knockdown emerges as a promising therapeutic strategy for enhancing axonal regeneration after SCI, because substantial evidence confirms that PTEN knockdown significantly augments intrinsic axonal regenerative capacity post-SCI [6,7]. Preclinical studies also demonstrate that neuron-specific PTEN knockdown reactivate mTOR signaling, thereby driving robust regenerative axon growth in vitro and in vivo [8]. Such regenerative process is mediated through mTOR-dependent mechanisms including up-regulation of protein synthesis, elevation of growth cone dynamics, and modulation of reactive glial environment. However, restoring locomotion function constitutes a persistent hurdle, where successful recovery relies on not only axon extension but also guiding them to natural region downstream of injury sites [9,10].

Spinal cord consists of ascending and descending tracts with parallel structures, which are responsible for transmitting signals between brain and peripheral tissue [11]. Unfortunately, regenerated axons often exhibit multi-directional projections, potentially resulting in neurite dysfunction [12–14]. Techniques such as electrospinning or 3-dimensional (3D) printing have been used to create stem cell-embedding scaffolds for promoting axonal extension [15–18]. For example, Koffler et al. [19] constructed a 3D-printed structure with channels filled with neuron stem cells, which effectively induced parallel axonal extension and improved the behavior of SCI animals. Li et al. [20] developed a biocompatible bioink consisting of functional materials with neuronal stem cells for 3D printing and then utilizing it in SCI repair. Wang et al. [21] arranged a layer-by-layer deposition of Schwann cells and mesenchymal stem cells to mimic organization presented in spinal cord organoids by 3D printing. Although individual therapeutic strategies showed potential, each strategy had significant limitations when applied in isolation. 3D-printed scaffolds alone provided essential physical guidance for axonal regrowth, yet they were fundamentally passive constructs. They failed to actively address the inhibitory molecular barriers, such as myelin-associated inhibitors, or the hostile, inflammatory post-injury microenvironment characterized by reactive astrocytes and proteoglycans [22]. Conversely, PTEN knockdown is a powerful molecular intervention that enhances the intrinsic regenerative capacity of neurons by promoting mTOR activity, effectively overcoming the neuron’s own reluctance to regenerate. However, this approach alone does not provide the critical structural support required for directed axonal regrowth across the lesion, nor does it modulate the inhibitory extracellular milieu that remains a formidable barrier [23]. Therefore, combining 3D-printed topological guidance with intrinsic axonal extension ability offers a promising solution for SCI.

Extracellular matrix (ECM) provides structural support and bioactive cues for promoting axonal regeneration [24,25]. As a crucial ECM component in the spinal cord, laminin is capable of enhancing axon growth and guiding axonal extension by influencing the Ephrin/Eph signaling pathway [26]. The laminin-derived IKVAV peptide with a motif from α1 chain of laminin-1 has been demonstrated to spatiotemporally control neurogenesis and direct neurite outgrowth to desired positions [27,28]. Self-assembling RADA_4_ peptide with a stable secondary structure serves as an ideal scaffold for cell migration and axonal regrowth [29]. Furthermore, peptide hydrogels can be engineered to optimize the extracellular microenvironment by presenting bioactive epitopes to promote cell adhesion and sequester inflammatory signals, creating a more permissive local environment [30]. Naturally, it inspires us to fabricate a novel peptide hydrogel by combining RADA_4_ and IKVAV peptides, which may optimize the injury microenvironment and stimulate intrinsic axonal regeneration by providing bioactive sites or modulating the Ephrin/Eph pathway in vivo.

**Fig. 1.**
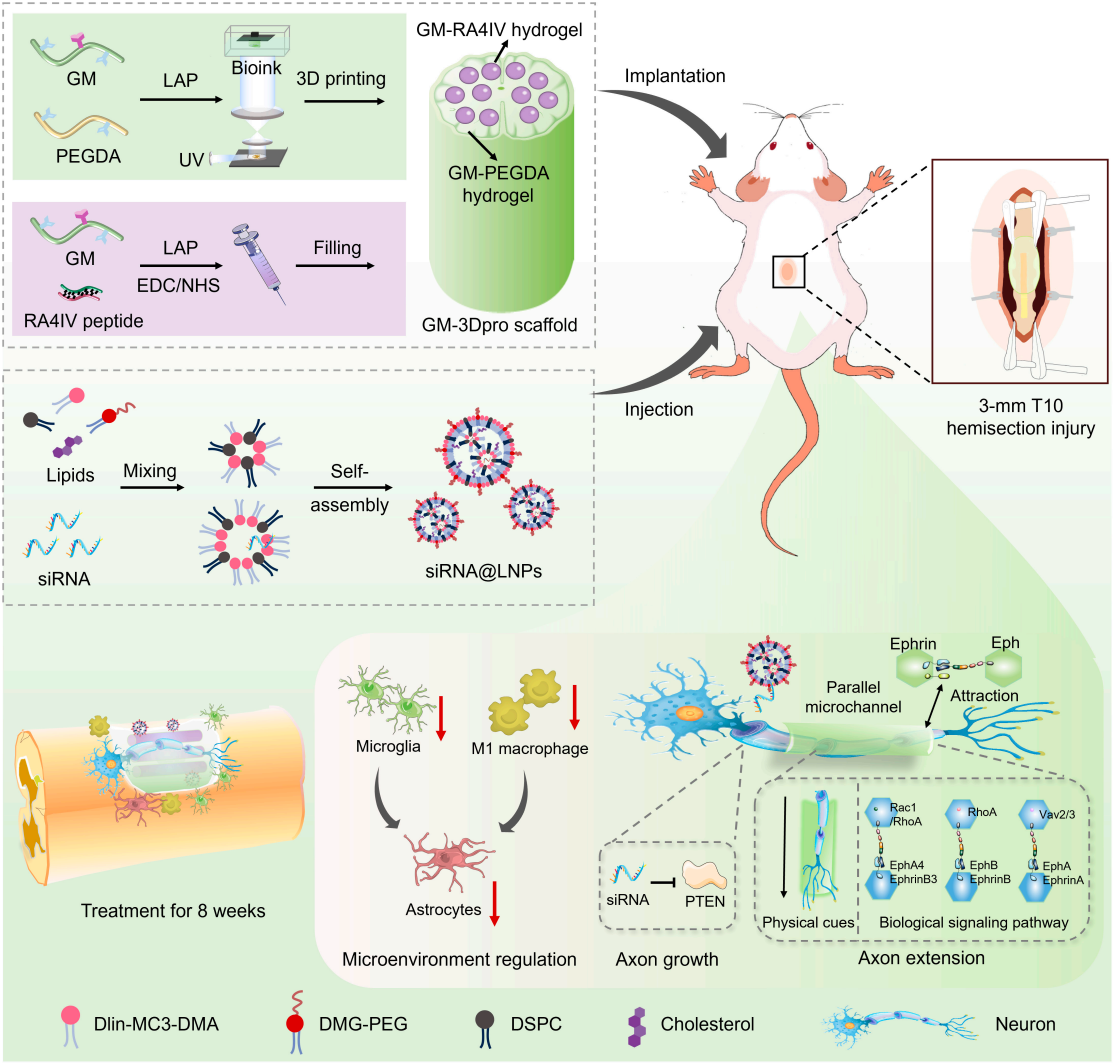
Schematic illustration showing fabrication of 3D-printed multi-channel scaffold incorporated with siRNA delivery system as well as their promotion effect on axonal regeneration for SCI.

In this study, we developed an integrated multichannel 3D-printed scaffold and small interfering RNA (siRNA) delivery system to synergistically promote spinal cord regeneration. This approach modulated tissue microenvironment, enhanced the intrinsic regenerative capacity of axons, and provided topographical guidance for directional axonal extension (Fig. 1). The bioactive scaffold included 2 distinct hydrogel components. On the one hand, a gelatin methacryloyl-poly(ethylene glycol) diacrylate (GM-PEGDA) hydrogel served as the primary architecture of scaffold, which was designed to reinforce mechanical properties and provide axonal directional guidance for axon regeneration through parallel microchannels. On the other hand, a gelatin methacryloyl-RADA_4_-IKVAV (GM-RA4IV) hydrogel was incorporated to fill these microchannels, where it functioned to protect residual tissues and create a favorable regeneration environment by suppressing post-injury neuroinflammation effectively. Furthermore, liposome nanospheres loaded with siRNA (siRNA@LNPs) were successfully delivered and achieved PTEN knockdown at the site of SCI. Extensive in vitro and in vivo data demonstrated that this strategy effectively guided the parallel axonal growth across the lesion site and up-regulated genes associated with the Ephrin/Eph signaling pathway, which precisely oriented the extending axons toward their native target regions. Importantly, the combination of gene knockdown with the 3D-printed scaffold significantly promoted the recovery of motor function in rats after SCI, particularly in regulating knee joint movement, gait, and body support. In all, these findings collectively demonstrated critical importance of enhancing intrinsic axonal regenerative capacity and guiding parallel axonal growth for neural circuit reconstruction, establishing a promising approach for functional recovery following SCI.

## Results

### Fabrication and characterizations of GM-RA4IV hydrogel

**Fig. 2.**
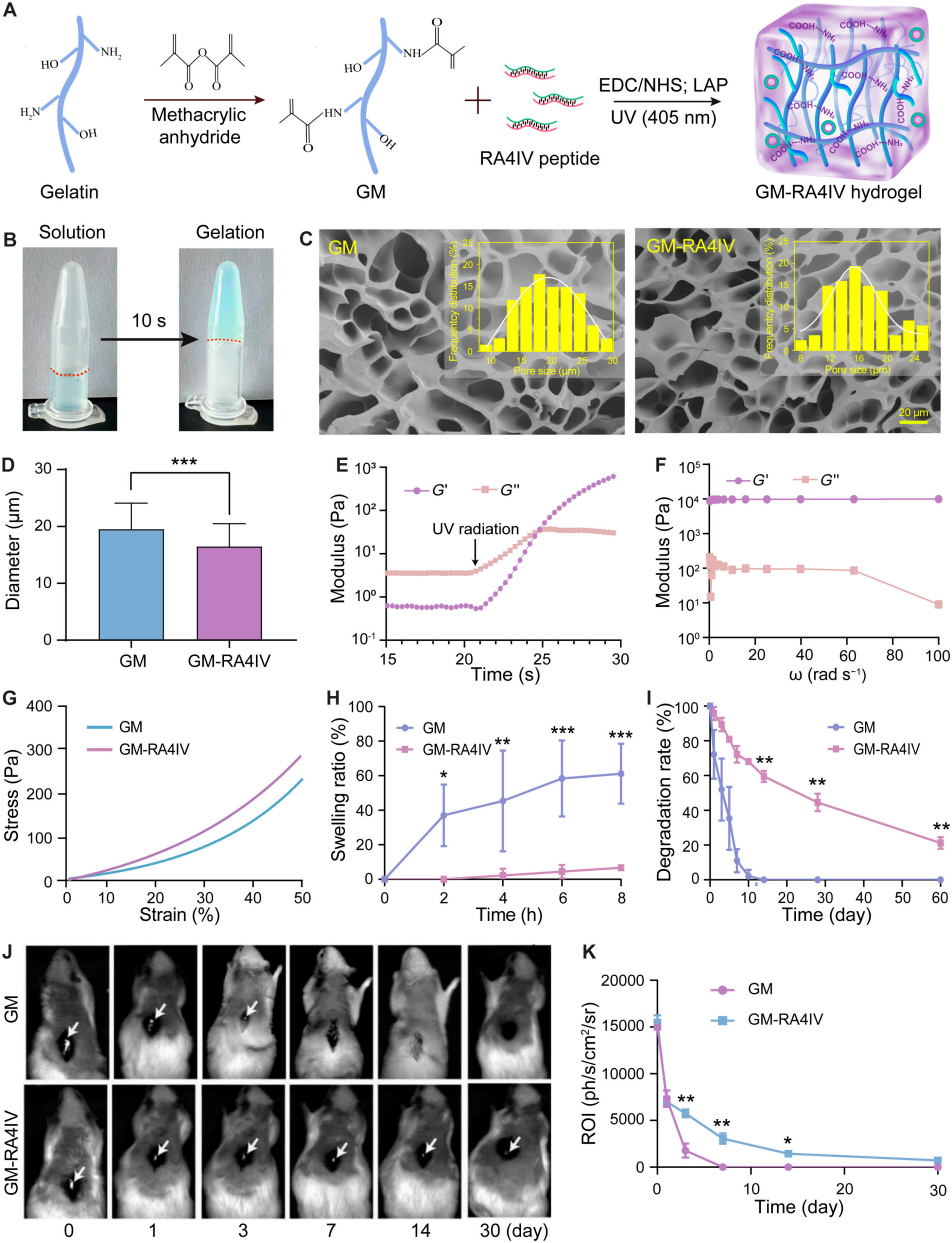
Basic physical properties of GM-RA4IV hydrogel. (A) Fabrication of GM-RA4IV hydrogel. (B) Sol–gel transition of GM-RA4IV after UV exposure. (C) SEM images and pore-size distributions of GM and GM-RA4IV hydrogels. (D) Average pore diameter based on SEM images (*n* = 3). (E) Rheological properties of GM-RA4IV solution upon UV-initiated polymerization. (F) Frequency sweep measurement of GM-RA4IV hydrogel. (G) Compressive stress–strain curves of GM and GM-RA4IV hydrogels. (H) Swelling and (I) degradation ratios of GM and GM-RA4IV hydrogels during 2 months in vitro (*n* = 3). (J) Fluorescence images and (K) quantification of region of interest (ROI) of GM and GM-RA4IV hydrogels about degradation during a period of 1 month in vivo (*n* = 3). Images in same roll were from same animal. All statistical data are represented as mean ± SD (*P < 0.05, **P < 0.01, ***P < 0.001). *T* test was used in (D), (H), (I), and (K).

A photocurable prepolymer (gelatin methacryloyl, GM) was synthesized from gelatin and methacrylic anhydride. The RADA_4_-IKVAV (RA4IV) peptide was then grafted onto GM prepolymer by a reaction between carboxyl groups and amino groups, resulting a ultraviolet (UV)-curable hydrogel (GM-RA4IV; Fig. 2A). We next evaluated physical properties of the GM-RA4IV hydrogel in vivo and in vitro. As shown in Fig. 2B, the GM-RA4IV solution rapidly crosslinked and formed a stable hydrogel upon exposure to UV irradiation (405 nm) for 10 s, as evidenced by its stability upon inversion of centrifuge tube. Subsequently, scanning electron microscopy (SEM) images of the freeze-dried hydrogels in Fig. 2C confirmed that all samples possessed highly porous architectures. The average pore diameters of the GM and GM-RA4IV hydrogels were measured to be 19.46 ± 4.64 μm and 16.44 ± 4.03 μm, respectively (Fig. 2D). Notably, the GM-RA4IV hydrogel displayed a more compact and homogeneous porous structure compared to the GM hydrogel. This increased structural uniformity was likely attributable to the additional crosslinking points introduced by the amide bonds formed between the RA4IV peptide and the GM prepolymer. Such a uniform porous network facilitated nutrient diffusion, metabolic waste removal, and formation of neovascularization, thereby playing a crucial role in supporting nerve regeneration [31, 71]. Rheological tests were further performed to examine the storage modulus (*G*′) and loss modulus (*G*″) profiles of the GM-RA4IV hydrogel. An intersection of the *G*′ and *G*″ curves occurring at 5-s post-UV exposure indicated a rapid gelation rate, confirming suitability of the GM-RA4IV solution as a bioink for light-based 3D printing (Fig. 2E). After complete crosslinking, frequency sweep test showed that *G*′ was greater than *G*″ and remained independent of frequency, indicating that the GM-RA4IV hydrogel maintained a stable structure under oscillatory shear deformation (Fig. 2F). Moreover, a compression test was conducted to evaluate mechanical properties of the GM-RA4IV hydrogel (Fig. 2G). An average compression strength of the GM-RA4IV hydrogel was 284.67 ± 12.83 Pa, significantly higher than that of the GM hydrogel (Fig. S1). This mechanical integrity of both GM and GM-RA4IV has been validated to maintain an integrated 3D structure, and the enhanced strength of GM-RA4IV will provide longer support for promoting spinal cord regeneration [13,32].

Regarding swelling behavior, the GM-RA4IV hydrogel maintained a low swelling ratio of less than 10%, in contrast to rapid expansion of volume observed in the GM hydrogel (Fig. 2H). The reduced swelling ratio also was attributed to the crosslinking of carboxyl and amine groups by 1-(3-dimethylaminopropyl)-3-ethylcarbodiimide hydrochloride/*N*-hydroxy succinimide (EDC/NHS). Newly formed covalent bonds acted as physical anchors, reducing the mobility and flexibility of the polymer chains. This created a tighter, more constrained network with smaller mesh sizes. As a result, the hydrogel had a reduced capacity to absorb and hold water molecules within its structure [33]. This low swelling property prevented excessive compression of damaged tissue area caused by water absorption and expansion of scaffold following implantation. Proper degradation behavior is crucial for facilitating the integration of scaffold with host tissue [34]. Specifically, new tissue gradually fills the degrading scaffold′s framework as the biomaterial degraded, facilitating formation of functional tissue interface and enhancing integration strength. Therefore, degradation kinetics of the hydrogels were systematically studied in vitro and in vivo. As shown in Fig. 2I, the GM-RA4IV hydrogel preserved approximately 50% of its original mass after 4 weeks of immersion in vitro, with the residual mass percentage further decreasing to 21.23% ± 2.72% by week 8, indicating a gradual degradation trend over time. To more accurately evaluate degradation behavior in vivo, fluorescent microspheres were embedded into the hydrogels and monitored using an imaging system. Results demonstrated that the GM hydrogel degraded completely within 7 d in vivo, whereas the Gel-RA4IV hydrogel exhibited a slower degradation rate, persisting for approximately 1 month (Fig. 2J and K). This discrepancy in degradation behavior was likely attributable to the use of EDC/NHS crosslinkers in the GM-RA4IV hydrogel. This property not only offered critical structural support but also gradually generated space that supported axonal regeneration and spinal cord tissue formation [13,35]. In conclusion, these physical results showed that the proposed GM-RA4IV hydrogel possessed suitable mechanical strength, uniform pore structure, low swelling rate, and appropriate biodegradation behavior.

### Biocompatibility and axonal outgrowth in vitro

**Fig. 3.**
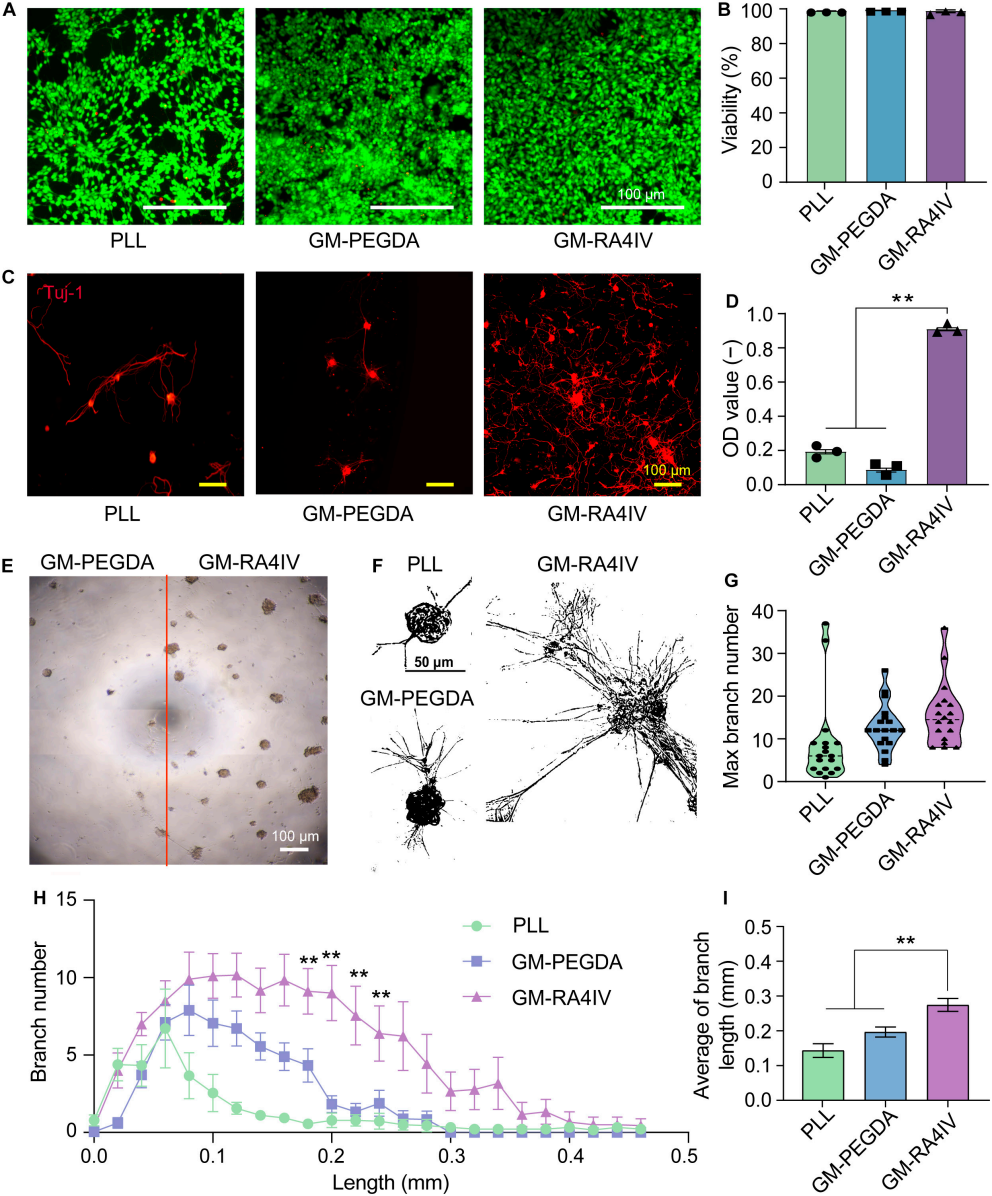
Biocompatibility and axonal outgrowth in vitro. (A) Live/Dead staining images and (B) quantification of viability of PC12 cells cocultured with PLL, GM-PEGDA, and GM-RA4IV hydrogels for 24 h (*n* = 3). (C) Tuj-1 immune staining images of DRG neurons cocultured with PLL-, GM-PEGDA-, and GM-RA4IV-coated plates for 24 h. (D) OD values of DRG neurons incubated in 3 conditions (*n* = 3). (E) Observation of DRG neurons grown in GM-PEGDA and GM-RA4IV half-coated plate. (F) Representative images of axonal extension of DRG neurons cocultured with PLL, Gel-PEGDA, and GM-RA4IV coating plates for 5 d. Quantitative analysis of (G) maximum branch numbers and (I) average length of axon branches in different conditions (*n* = 18). (H) Quantification of branch number of axons in different lengths (*n* = 3). All statistical data are represented as mean ± SD (**P < 0.01). One-way ANOVA followed by Tukey’s test was used in (D), (H), and (I).

Excellent biocompatibility is a condition for clinical application of SCI repair. Live/Dead staining assay was applied to evaluate biocompatibility of the hydrogels. PC12 cells, known for their stability and consistency, were cocultured with the poly-l-lysine (PLL), GM-PEGDA, and GM-RA4IV samples for 24 h. Notably, PLL promotes neuronal differentiation, maturation, and network formation, and therefore serves as a positive control [36]. As shown in Fig. 3A, green fluorescence intensity observed in the GM-PEGDA and GM-RA4IV hydrogels was comparable to that of the PLL group. Quantitative analysis further confirmed that the cell survival rate of these groups both exceeded 98%, which fully demonstrated that neither the GM-PEGDA nor GM-RA4IV hydrogel exhibited significant cytotoxicity (Fig. 3B). Collectively, these findings in vitro indicated that the fabricated GM-RA4IV hydrogel exhibited excellent cytocompatibility, warranting its further evaluation in vivo as a promising scaffold for spinal cord repair.

Neurite outgrowth and complexity are critical indicators of neuronal maturation and signal transmission capacity [37]. To assess potential of the GM-RA4IV hydrogel to promote neuronal differentiation and neurite extension, dorsal root ganglion (DRG) neurons were cocultured in the PLL-, GM-PEGDA-, and GM-RA4IV-coated 96-well plates for 24 h. Immunofluorescence staining for neuron-specific microtubule element marker β-III-Tubulin (Tuj-1) was performed. As confirmed in Fig. 3C, significantly enhanced neurite outgrowth and network complexity were observed exclusively in the GM-RA4IV group, but not in the pure synthetic polymer control (GM-PEGDA), a result directly attributable to the bioactivity of the RA4IV peptide. Furthermore, the GM-RA4IV group showed a higher optical density (OD) value (0.96 ± 0.02), as measured by the cell counting kit-8 (CCK-8) assay (Fig. 3D). These results confirm that the GM-RA4IV hydrogel incorporating the RADA_4_-IKVAV peptide successfully enables peptide presentation, preserves bioactivity, and significantly enhances both cell proliferation and neurite density. To directly evaluate effects of the hydrogels on neural growth, DRG neurons were seeded in a plate divided into 2 halves: one coated with the GM-PEGDA hydrogel and the other with GM-RA4IV hydrogel (Fig. 3E). Obviously, the GM-RA4IV-coated side supported obviously greater neuronal adhesion and neurite density than the GM-PEGDA-coated side, underscoring superior neuropermissive properties of the GM-RA4IV hydrogel. After 5 d of coculturing, neurite extension was observed under all conditions. However, DRG neurons grown in the GM-RA4IV-coated plate displayed markedly longer and more extensively branched neurites compared to those in the PLL and GM-PEGDA groups (Fig. 3F). Quantitative analysis confirmed that neurons in the GM-RA4IV sample exhibited a 1.2- and 2.4-fold increase in the maximum branch number compared to the GM-PEGDA and PLL coating, respectively (Fig. 3G). Assessment of neuronal complexity using Sholl analysis revealed that the DRG neurons cultured in the GM-RA4IV group possessed a higher number of branches within 0.08 to 0.24 mm from the cell soma relative to the other groups (Fig. 3H). Additionally, average neurite branch length in the GM-RA4IV group was 0.27 ± 0.08 mm, significantly greater than that in the GM-PEGDA (0.20 ± 0.06 mm) or PLL groups (0.14 ± 0.08 mm; Fig. 3I). To gain insight into molecular mechanisms underlying the enhanced neuronal growth and complexity observed with the GM-RA4IV sample, real-time quantitative polymerase chain reaction (RT-PCR) analysis was conducted. This result revealed a significant up-regulation in the expression of Epha3 (a gene implicated in axonal guidance and synaptic plasticity) in neurons cultured in the GM-RA4IV-coated plate compared to the GM-PEGDA group (Fig. S2). These data demonstrated that the GM-RA4IV hydrogel provided a superior, permissive substrate for axon adhesion and growth compared to the GM-PEGDA hydrogel. This preferential growth within the GM-RA4IV material was the fundamental basis for its role as an axon guidance cue when structured into parallel channels within the 3D-printed scaffold. In summary, these findings supported the hypothesis that hydrogels functionalized with the laminin-derived IKVAV peptide promoted robust neurite outgrowth and complexity, potentially mediated through the Ephrin/Eph signaling pathway [38,39].

### GM-RA4IV protected residual tissue and promoted axon regeneration in vivo

**Fig. 4.**
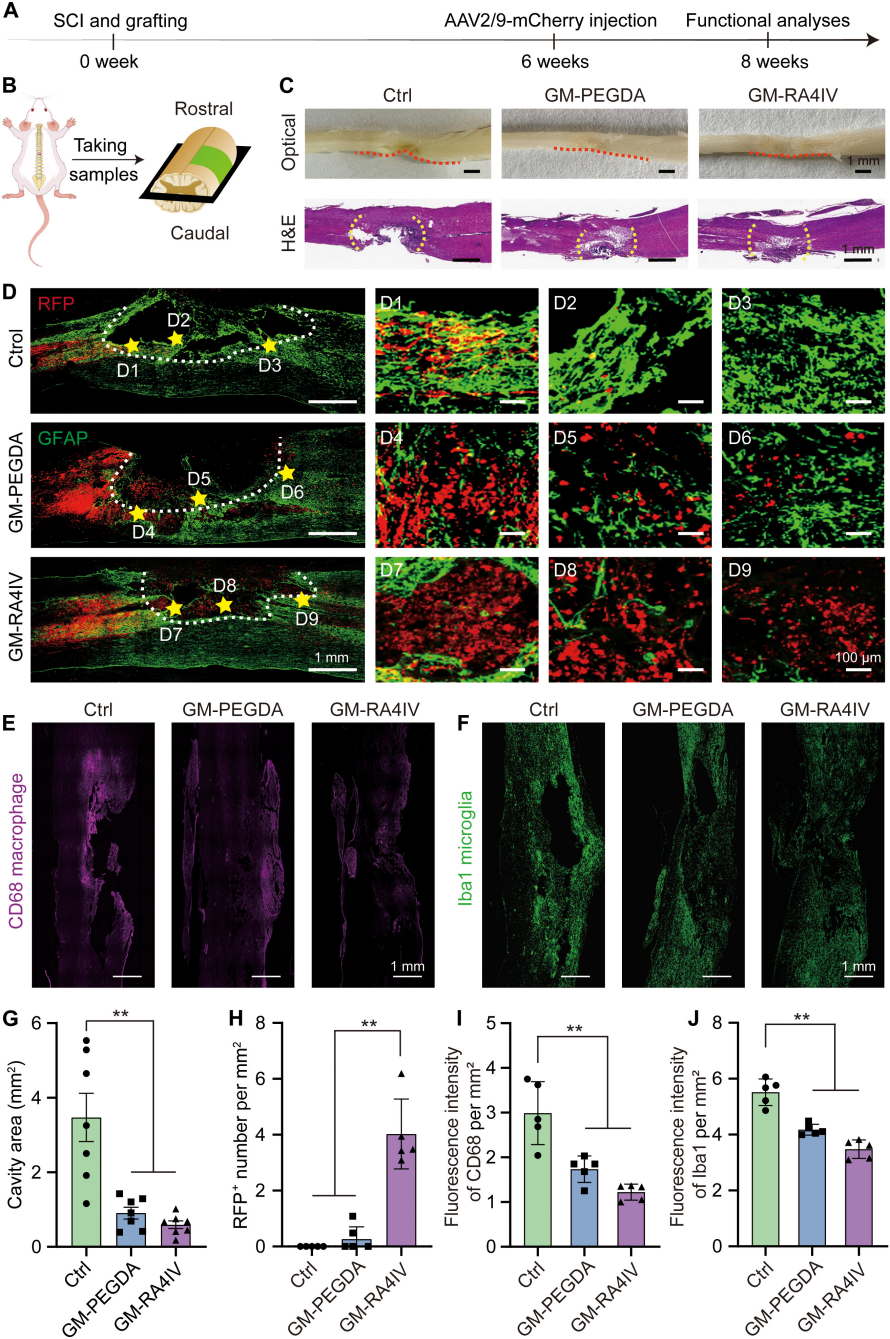
GM-RA4IV hydrogel protected residual tissue and promoted axon regeneration in vivo. (A) Experimental timeline. (B) Preparation of longitudinal slices. (C) Optical and H&E staining images of injured spinal cord after 8 weeks. Red and yellow dotted lines indicate edges of spinal tissue and damaged sites, respectively. (D) Representative immunofluorescence staining and magnified images of sagittal sections for GFAP (green)- and AAV2/9-mCherry (red)-traced axons under different conditions after 8 weeks. White dotted lines indicate edges of SCI, and yellow pentagons represent enlarged position. Immunofluorescence staining images of (E) CD68 and (F) Iba1 in 3 groups at 8 weeks post-injury. (G) Quantitative analysis of cavity areas in Ctrl, GM-PEGDA, and GM-RA4IV groups (*n* = 7). (H) Quantification of axon number at injury site per mm^2^ (*n* = 5). Fluorescence intensities of (I) CD68 and (J) Iba1 per area in different groups (*n* = 5). All statistical data are represented as mean ± SD (**P < 0.01). One-way ANOVA followed by Tukey’s test was used in (G) to (J).

Having established neuritogenesis potential of the GM-RA4IV hydrogel in vitro, we next evaluated its efficacy in promoting functional recovery in vivo using a rat model of SCI. This assessment focused on its ability to protect residual tissue and promote axon regeneration. Experimental timeline was schemed in Fig. 4A. Specifically, a T10 hemisection injury with a length of 3 mm was established in rats [40]. Lesion cavity was subsequently filled with either the GM-PEGDA or GM-RA4IV hydrogel. To trace growth and projection, we used AAV2/9-mCherry, a serotype that specifically infected neurons in vivo. It was injected into healthy segments at 6 weeks post-injury to label axons projecting toward the lesion site [41]. Spinal cord tissue samples were collected 8 weeks post-surgery for histochemical analysis and immunofluorescence staining (Fig. 4B). Initial macroscopic examination revealed that spinal cord deformations in the GM-PEGDA- and GM-RA4IV-treated rats were less pronounced compared to the Ctrl group. Consistent with this observation, hematoxylin and eosin (H&E) staining of transverse tissue sections demonstrated a significant reduction in cystic cavity formation at the lesion epicenter in the hydrogel implantation groups (Fig. 4C). In addition, a series of sequential H&E-stained sections were used to quantify cavity areas. As shown in Fig. 4G, cavity areas at injured sites of the GM-PEGDA and GM-RA4IV groups were reduced to 40% and 26% of that in the Ctrl group, respectively. This reduction can be primarily attributed to the physical support provided by the hydrogels. Specifically, the hydrogels filled the cavity structures formed during phase of injury, thereby imparting mechanical stability to the surrounding residual tissue and preventing further cavity expansion and coalescence. Together, these findings indicated that hydrogel implantation conferred protective effects on peri-lesional tissues by mitigating mechanical destabilization and reducing cavity progression. These data suggested that transplantation of the hydrogels not only enhanced protection for surrounding tissues but also supported regenerative processes within damaged spinal cord.

To assess neural regeneration across treatment groups comprehensively, we performed immunofluorescence staining. Red fluorescent protein (RFP) was visualized to AAV2/9-mCherry-labeled axons, while glial fibrillary acidic protein (GFAP) staining identified activated astrocytes (Fig. 4D). Overall observational results revealed that the GM-RA4IV group exhibited a reduced lesion area and formation of new tissue within the lesion cavity compared to the GM-PEGDA and Ctrl situations. To gain deeper insight into axonal regeneration, we analyzed magnified views of the proximal, middle, and distal regions of the injury site. The Ctrl group consistently displayed a high density of GFAP^+^ astrocytes across all segments, indicating significant formation of glial scar encircling the lesion. Conversely, the GM-RA4IV hydrogel showed a significant reduction in reactive astrocyte proliferation, a hallmark of secondary injury, and supported extensive ingrowth of axons (RFP^+^) into the lesion area. Additionally, 5-hydroxytryptamine (5-HT) axons have been shown to promote functional recovery after SCI by increasing neurotransmission and altering the motility of growth cones [42,43]. Thus, anti-5-HT and anti-neurofilament (NF200, indicating newly regenerated nerve fibers) were further stained. As shown in Fig. S3A and B, the GM-RA4IV scaffold exhibited greater densities of 5-HT and NF200 axons penetrating the lesion site compared to the GM-PEGDA group. Quantitative analyses of RFP^+^, 5-HT^+^, and NF200^+^ axons across all groups fully corroborated these observations, demonstrating a significant increase in regenerated axons associated with the GM-RA4IV implantation (Fig. 4H and Fig. S3C and D). These findings indicated that the GM-RA4IV hydrogel enhanced histological recovery after SCI by mitigating glial scar formation and robustly promoting the regeneration of new axonal bundles. This experiment demonstrated that the GM-RA4IV hydrogel provides a significantly more permissive substrate for neuronal adhesion and axonal growth than the GM-PEGDA hydrogel. This inherent bioactivity is the fundamental mechanism that allows the GM-RA4IV hydrogel to act as an active guidance conduit. For future study in 3D printing structure, axons preferentially extend within the GM-RA4IV-filled channels, thereby being guided along the defined parallel architecture of the scaffold.

Neuroinflammation, a key driver of secondary damage after SCI, is characterized by the activated microglia and infiltration of pro-inflammatory (M1) macrophages. Modulating this response is critical for neuroprotection and functional recovery [44]. Therefore, we evaluated the immunomodulatory effects of the GM-RA4IV hydrogel using markers for M1 macrophages (CD68) and microglia (Iba1). Figure 4E shows that fluorescence brightness in the Ctrl group was higher than that of the GM-PEGDA and GM-RA4IV situations. Fluorescence intensities of CD68 by quantitative analysis were 2.99 ± 0.63, 1.73 ± 0.26, and 1.22 ± 0.16 per mm^2^ in the Ctrl, GM-PEGDA, and GM-RA4IV groups, respectively, which indicated the inflammation level of hydrogel-mediated reduction (Fig. 4I). Similarly, Iba1 staining demonstrated markedly reduced level of microglial activation in hydrogel-treated animals, with the GM-RA4IV group showing significant suppression (Fig. 4F and J). These data indicated the GM-RA4IV hydrogel effectively attenuated the inflammatory response at the lesion sites. In conclusion, the GM-RA4IV hydrogel effectively prevented the enlargement of lesion sites and promoted axonal regeneration, which benefited from dual mechanisms of the grafted RA4IV peptide. On the one hand, the RA4IV peptide regulated the injury microenvironment, especially by inhibiting harmful neuroinflammation, thereby protecting spared tissues and creating a more favorable environment for regeneration [45,46]. On the other hand, it directly provided neurotrophic support to enhance adhesion, growth, and maturation of nerve cells [47].

### Immune reaction remission and positive locomotion revealed by RNA sequencing

RNA sequencing analysis provided valuable molecular insights into hydrogel-mediated spinal cord repair mechanisms. A total of 124 differentially expressed genes (DEGs) were identified between the Ctrl and hydrogel implantation groups (GM-PEGDA or GM-RA4IV) based on a heatmap (Fig. S4A). Notably, several pro-inflammatory genes, including Tnfrsf9, Ifit3, Cxcl17, Cx3cr1, Gpr84, and C1qa, exhibited high expression in the Ctrl group compared to the hydrogel implantation conditions. This finding corroborated our immunofluorescence data in vivo, further suggesting that the implantation of hydrogels effectively inhibited inflammatory cascade reaction and regulated the endogenous microenvironment after SCI.

Gene Ontology (GO) pathway enrichment analysis revealed correlations between RA4IV peptide and spinal cord regeneration, including biological processe (BP), cellular component (CC), and molecular function (MF). As shown in Fig. S4B, the GM-PEGDA group significantly enriched in signaling pathways related to regulation of stimulus response, immune system processes, and multicellular space, compared with the Ctrl group. Conversely, DEGs in the GM-RA4IV situation showed distinct enrichment in positive regulation of locomotion, cascade regulation of mitogen-activated protein kinase (MAPK), and regulation of ion transport and cell adhesion. The RA4IV peptide engaged cell surface integrins via its specific binding motif, triggering intracellular signaling cascades. This process dynamically regulated focal adhesion assembly/disassembly at the cell–ECM interface, thereby facilitating cell migration [48]. It was crucial that MAPK pathway components critically modulated local inflammation in SCI, mainly by suppressing inflammatory mediator release and mitigating neuronal apoptosis [49]. Further comparative analysis revealed that the GM-RA4IV group specifically enhanced enrichment of cell migration and locomotor regulation pathways relative to the GM-PEGDA hydrogel. These findings demonstrated that the RA4IV peptide enhanced the therapeutic effect of hydrogel system on SCI by facilitating cellular migration, suppressing neuroinflammation, and promoting motor function recovery. It was worth noting that axonal regeneration-associated genes showed no differential expression among the 3 groups. This suggested that the peptide-functionalized hydrogels primarily rescued spared axons rather than induced strong regeneration. Consequently, the observed functional recovery after SCI in vivo may be attributed to neuroprotection and plasticity of surviving neural circuits. These results highlighted the need for combinatorial strategies to enhance axon regeneration and promote robust functional recovery following SCI.

### Improved axon extension by PTEN knockdown

**Fig. 5.**
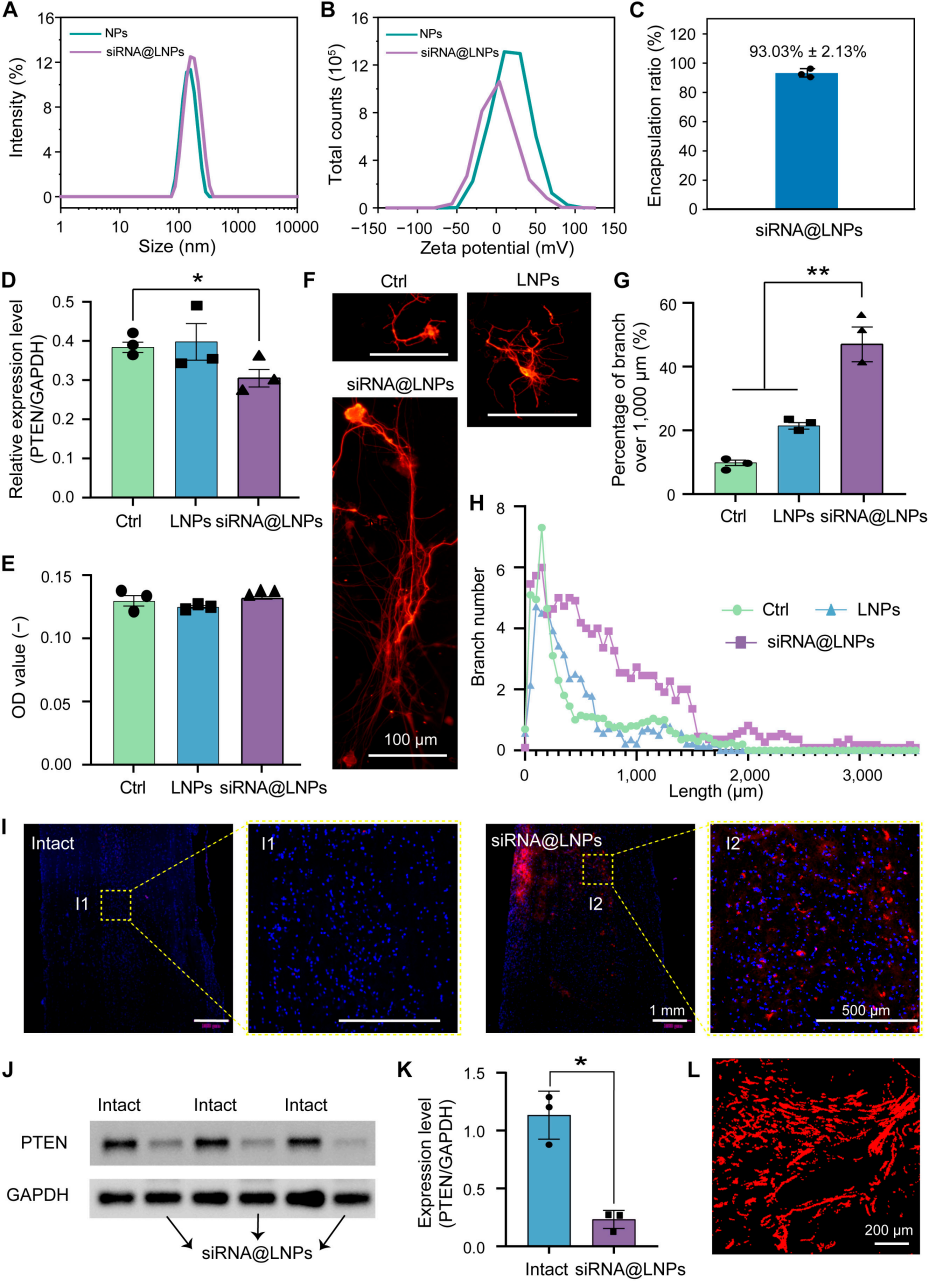
siRNA@LNPs down-regulated expression level of PTEN and improved axon extension in vitro and in vivo. (A) Particle size and (B) zeta potential of LNPs and siRNA@LNPs. (C) Encapsulation ratio of siRNA (*n* = 3). (D) Relative expression level of PTEN in Ctrl, LNPs, and siRNA@LNPs groups (*n* = 3). (E) OD values of DRG neurons cocultured in 3 conditions for 24 h after siRNA transfection (*n* = 3). (F) Tuj-1 staining images of DRG neurons in different conditions. (G) Percentage of long axon branches over 1,000 μm quantified dependent on Tuj-1 staining images (*n* = 3). (H) Branch numbers of different lengths ranging from 0 to 3,500 μm for 3 conditions. (I) Fluorescence signals of siRNA delivery in Intact and siRNA@LNPs groups. Images on the right are magnified version of yellow quadrilateral on the left. (J) Western blot analyses presenting expression level of PTEN at 2 weeks after siRNA@LNPs injection (*n* = 3). (K) Quantification of expression level of PTEN based on Western blotting images (*n* = 3). (L) Representative image of axonal regeneration in the siRNA@LNPs + GM-RA4IV group at 8 weeks post-injury. All statistical data are represented as mean ± SD (*P < 0.05, **P < 0.01). One-way ANOVA followed by Tukey’s test was used in (D), (G), and (K).

Despite the complexity of SCI regeneration mechanisms, critical signaling pathways in neural cells offer promising therapeutic siRNA targets. PTEN knockdown plays a critical role in regulating axonal regeneration by reactivating the mTOR signaling pathway [50–52]. Therefore, to leverage this mechanism, the siRNA@LNPs was successfully prepared using a microfluidic technology and characterized for key physical properties. As shown in Fig. 5A, average particle sizes of the LNPs and siRNA@LNPs were 138.59 ± 9.22 and 167.17 ± 45.07 nm, respectively. However, zeta potential of the siRNA@LNPs was 8.04 ± 7.04 mV, lower than that of the LNPs (16.47 ± 6.25 mV), mainly due to negative charge of the siRNA (Fig. 5B). In addition, encapsulation efficiency of siRNA reached 93.03% ± 2.13% as determined by a concentration standard curve quantification (Fig. 5C and Fig. S5). In summary, these results indicated that the siRNA@LNPs have an appropriate nanoscale dimension, neutral surface charge, and high siRNA loading capacity.

Effective release of siRNA is crucial for improving therapeutic effect and promoting neurological function recovery. We evaluated the siRNA release efficiency and knockdown function by RT-PCR. Expression levels of PTEN in the Ctrl, LNPs, and siRNA@LNPs groups were 0.37 ± 0.01, 0.41 ± 0.05, and 0.32 ± 0.02, respectively, indicating that the liposome-encapsulated siRNA achieved effective release while maintaining knockdown function (Fig. 5D). CCK-8 assay further suggested no significant impact of PTEN knockdown on DRG neuron viability, thereby eliminating cytotoxicity concerns (Fig. 5E). To investigate effect of the PTEN knockdown on axonal regeneration, we performed immunofluorescence staining using the neuronal marker Tuj-1. As shown in Fig. 5F, DRG neurons treated with the siRNA@LNPs group exhibited obviously longer axonal branches than those in the Ctrl and LNPs conditions. Quantification of axon length distribution revealed that majority of axons in the Ctrl and LNPs groups ranged from 0 to 1,000 μm, while the PTEN knockdown condition induced a distinct shift of the distribution toward longer axons with a substantial proportion extending beyond 1,000 μm and up to 2,500 μm (Fig. 5H). The percentage of the axons exceeding 1,000 μm in the siRNA@LNPs group was significantly higher than other groups, demonstrating a promising efficacy of PTEN suppression in promoting axon outgrowth in vitro (Fig. 5G).

To validate these findings in vivo, we injected the siRNA@LNPs into the spinal cord and confirmed a successful siRNA localization at the injection site using 5-carboxyfluorescein (FAM) fluorescence (Fig. 5I). Western blot analysis was used to evaluate the expression level of PTEN at 2 weeks after injection. Compared with the Intact group (no treatment), PTEN protein bands in the siRNA@LNPs group showed a lower intensity, and the expression level of PTEN was quantitatively reduced by 70% (Fig. 5J and K). Next, we investigated the effect of PTEN knockdown on axon regeneration in the context of hydrogel grafting in vivo. Specifically, the siRNA@LNPs was injected into the target site. Two weeks after the siRNA injection, a spinal cord hemisection was performed at the same site, followed by transplantation of the GM-RA4IV hydrogel. According to the AAV2/9-syn-mCherry tracing, extensive regeneration of nondirectional axons into the hydrogel was observed (Fig. 5L). These findings highlighted the importance of siRNA delivery in regulating the expression level of PTEN protein for spinal cord repair, particularly in promoting axonal regeneration.

### siRNA@LNPs + GM-3Dpro group improved locomotion recovery in SCI rats

**Fig. 6.**
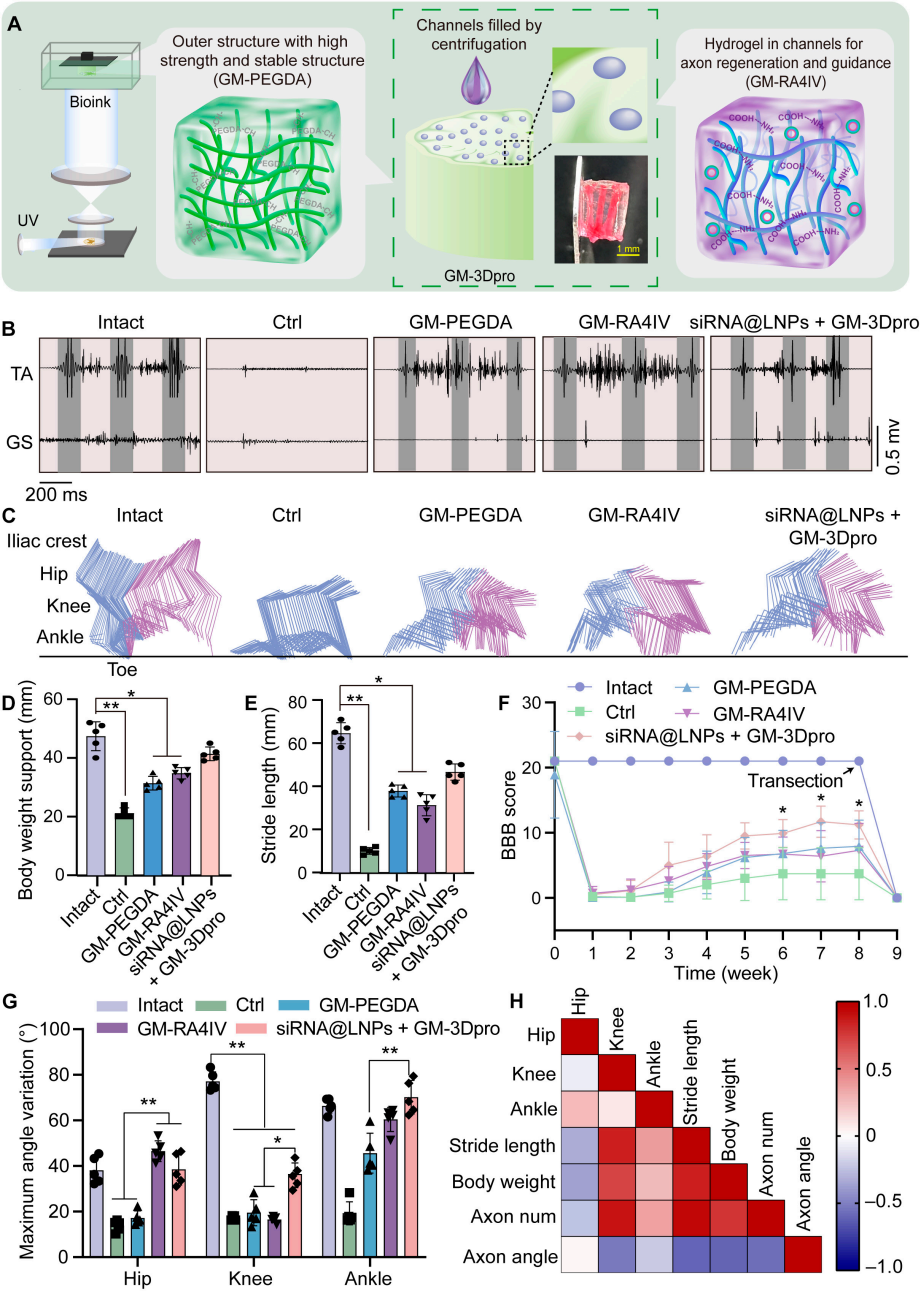
siRNA@LNPs + GM-3Dpro group reconstructed spinal cord circuits and promoted hind-limb locomotion function recovery of rats. (A) Schematic diagram of GM-3Dpro scaffold preparation and partial physical image. (B) EMG recording of TA and GS muscles and (C) color-coded stick views of kinematic hind-limb movement of Intact, Ctrl, GM-PEGDA-treated, GM-RA4IV-treated, and siRNA@LNPs + GM-3Dpro-treated groups. Quantitative analysis of (D) body weight support, (E) stride length, and (F) BBB scores weekly of 5 groups (*n* = 5). (G) Quantification of maximum angle variations of hip, knee, and ankle movements in free-walking animals (*n* = 5). (H) Correlation matrix of hip, knee, ankle movement, axon number, and axon projection orientation in Intact, Ctrl, GM-RA4IV, and siRNA@LNPs + GM-3Dpro groups. All statistical data are represented as mean ± SD (*P < 0.05, **P < 0.01). One-way ANOVA followed by Tukey’s test was used in (D) to (G).

After nerve injury, axons need to grow in the correct direction to reconstruct nerve connections and improve locomotion recovery. To provide directional topographical guidance cues for axonal growth, we utilized a high-resolution digital light projection 3D printing technology to construct the GM-PEGDA scaffold that exhibited excellent mechanical property and good biocompatibility (Fig. 3A and Fig. S6). Within this scaffold, parallel channels with a diameter of 200 μm were designed to exceed the size of axonal tracts, thereby providing sufficient space for axonal regeneration. Subsequently, the soft, conjugated GM-RA4IV hydrogel was infused into these microchannels to form the final composite scaffold (GM-PEGDA/GM-RA4IV, GM-3Dpro), which was capable of effectively promoting oriented axonal regeneration and functional recovery after SCI (Fig. 6A). We performed electromyography (EMG) to evaluate efficacy of the siRNA@LNPs + GM-3Dpro group in repairing spinal cord neural circuits and restoring motor function. EMG signals were recorded from tibialis anterior (TA) and gastrocnemius soleus (GS) muscles of free-walking animals (Fig. S7). As shown in Fig. 6B, EMG activity of TA muscle was presented in the GM-PEGDA, GM-RA4IV, and siRNA@LNPs + GM-3Dpro groups, resembling that in the Intact group. However, the amplitude of GS muscle in the siRNA@LNPs + GM-3Dpro group was observed to be slightly smaller than that in the Intact group. In stark contrast, minimal EMG activity was detected in both the TA and GS muscles of the Ctrl group.

To further evaluate the long-term therapeutic efficacy of the siRNA@LNPs + GM-3Dpro group on SCI, we performed detailed hindlimb kinematic analyses in freely ambulating animals, tracking the iliac crest, hip, knee, ankle, and toe. As expected, full stepping and complete paralysis were observed in the Intact and Ctrl groups, respectively. Notably, owing to the guidance from its parallel channels and PTEN knockdown, the siRNA@LNPs + GM-3Dpro group exhibited significantly improved motor function compared to the GM-PEGDA and GM-RA4IV scaffolds (Fig. 6C). As shown in Fig. 6D and E, rats in the GM-PEGDA and GM-RA4IV groups exhibited uncoordinated movements of knee and ankle joints, along with shorter stride length and reduced support of body weight compared to those in the Intact group, indicating a suboptimal stepping. In contrast, the siRNA@LNPs + GM-3Dpro group, benefiting from the orderly axonal regeneration guided by the parallel channels, displayed significantly greater body weight support and longer stride length, which correlated with the improved EMG activity in the TA and GS muscles. Hindlimb locomotion recovery was further assessed using the Basso–Beattie–Bresnahan (BBB) score. The BBB scores were significantly higher in the siRNA@LNPs + GM-3Dpro grafting group from 6 to 8 weeks post-injury (Fig. 6F). To determine whether the observed functional recovery depended on the presence of the implanted scaffolds, a subset of animals underwent a second transection at the graft site 8 weeks post-injury. This intervention abolished the functional improvements within 1 week, indicating that the recovery was mediated by the grafts. In detail, the GM-PEGDA and GM-RA4IV hydrogels promoted functional recovery by protecting tissue from secondary injury and providing a permissive matrix for axon regeneration. Moreover, the synergistic effect of 2 key features, the physical guidance of parallel channels and the pro-regenerative signaling via PTEN knockdown, in the siRNA@LNPs + GM-3Dpro group facilitated robust and well-aligned axonal regeneration and neural circuit reorganization, which enabled paralyzed rats to regain sustained capacity for weight-bearing stepping.

We further quantified joint kinematics by measuring the maximum angle variation in different conditions. Analysis revealed that the siRNA@LNPs + GM-3Dpro group significantly improved knee movement. Importantly, there was no significant difference in hip and ankle movements between the Intact and siRNA@LNPs + GM-3Dpro groups (Fig. 6G). Correlation coefficients were employed to evaluate relationship between locomotion behavior and regenerated axon in the Intact, GM-RA4IV, and siRNA@LNPs + GM-3Dpro conditions. According to the correlation matrix, the regenerated axon number and axon projection angle were positively and negatively correlated with the knee joint movement, stride length, and body weight, respectively (Fig. 6H). Therefore, grafting of the siRNA@LNPs + GM-3Dpro significantly improved locomotion recovery in SCI animals, especially playing a crucial role in regulating knee joint movement, stepping, and body support.

### Enhanced parallel axon projection by 3D-printed channel structure

**Fig. 7.**
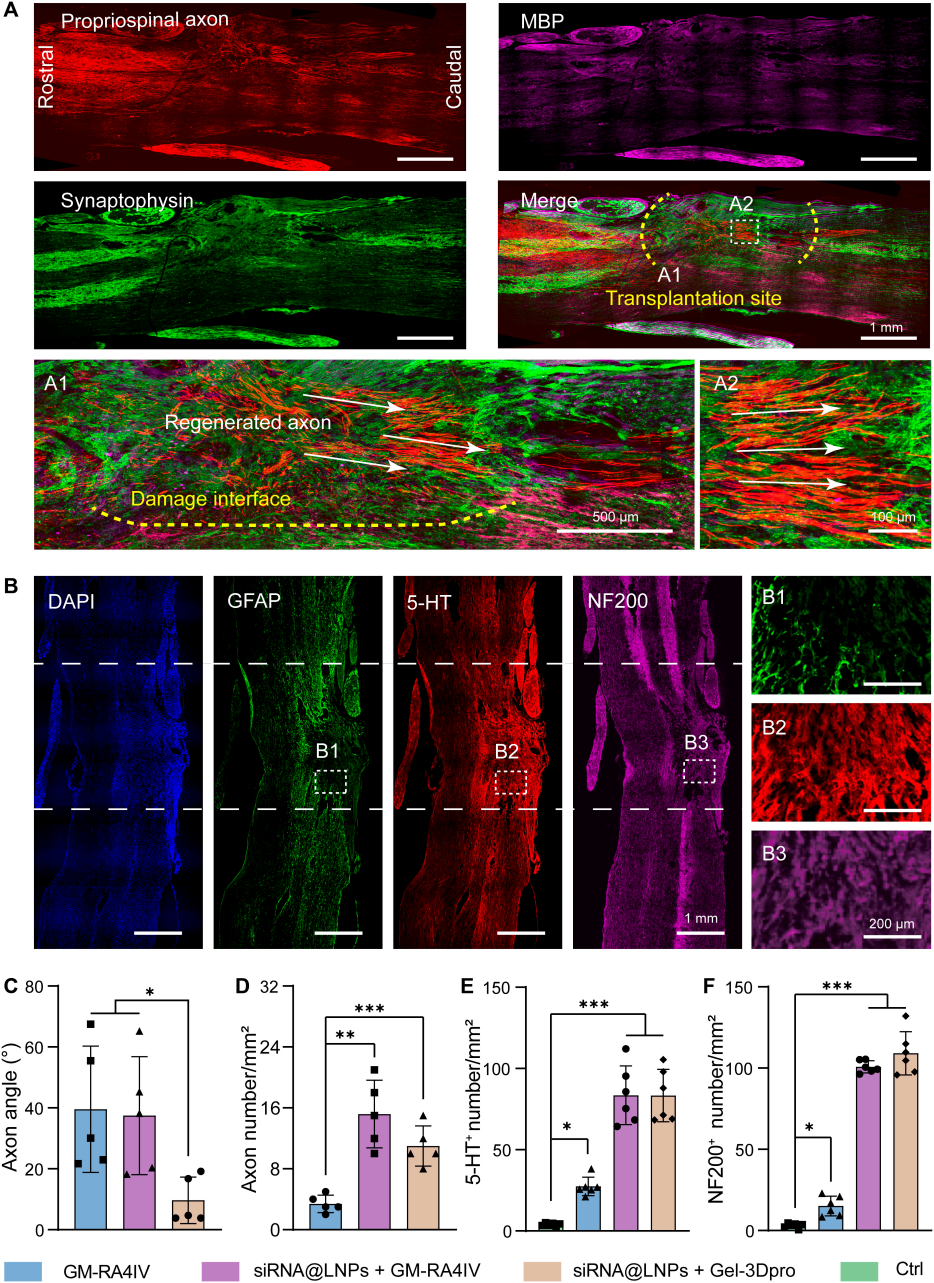
siRNA@LNPs + GM-3Dpro group induced parallel oriented axons regeneration in vivo. (A) AAV2/9-mCherry-traced axons and MBP (magenta)-/synaptophysin (green)-stained sagittal sections in the siRNA@LNPs + GM-3Dpro group. Enlarged images present longitudinally projected axons in (A1) long-distance and (A2) locally parallel projected axons. White arrow represents direction of axon extension. (B) Representative images of DAPI (blue), GFAP (green), 5-HT (red), and NF200 (magenta) at 12 weeks in the siRNA@LNPs + GM-3Dpro group. Right panels present higher magnifications of areas within white rectangles in left panels (B1 to B3), and white dashed lines present damaged areas. Quantification of (C) projection angle and (D) number of axons in GM-RA4IV, siRNA@LNPs + GM-RA4IV, and siRNA@LNPs + GM-3Dpro groups (*n* = 5). Quantitative analysis of (E) 5-HT^+^ and (F) NF200^+^ axon numbers per mm^2^ in 4 conditions (*n* = 6). All statistical data are represented as mean ± SD (*P < 0.05, **P < 0.01, ***P < 0.001). One-way ANOVA followed by Tukey’s test was used in (C) to (F).

In view of the highly efficient postoperative recovery of motor function in rats, we performed immunofluorescent staining with multiple specific markers at the lesion site to evaluate the scaffold’s ability for promoting parallel axon regeneration in vivo. As shown in Fig. 7A, treatment with the siRNA@LNPs + GM-3Dpro group promoted robust, long-distance axonal growth into distal tissues beyond the lesion site. Higher-magnification images of the injured regions (A1 and A2) showed that regenerated axons extended along a parallel trajectory relative to a reference axis spanning from the proximal to the distal boundary, guided by the channels of the 3D-printed scaffold. This orderly growth pattern was accompanied by an increase in red fluorescence intensity. Furthermore, representative immunostaining of myelin basic protein (MBP) and synaptophysin, markers of mature axons and synapses, confirmed myelination of regenerated axons and formation of new synapses. These findings suggested that the newly formed axons established functional connections with other neurons, representing an essential step toward functional recovery [53]. Quantitative analysis further demonstrated the efficacy of the guidance channels (Fig. 7C). The average angle of regenerated axons relative to the reference axis in the siRNA@LNPs + GM-3Dpro group (9.67° ± 6.82°) was significantly smaller than that in the GM-RA4IV (39.55° ± 18.53°) and siRNA@LNPs + GM-RA4IV (37.48° ± 17.31°) groups. This result indicated that the parallel microchannels of the GM-3Dpro scaffold effectively guided axonal regrowth. Moreover, the PTEN knockdown (siRNA@LNPs + GM-RA4IV and siRNA@LNPs + GM-3Dpro groups) resulted in a 2-fold increase in number of the regenerated axons compared to the GM-RA4IV group (Fig. 7D).

4′,6-Diamidino-2-phenylindole (DAPI) staining in Fig. 7B showed complete tissue integration of the grafted hydrogel, indicating a successful lesion repair. Given the pivotal role of astrocytes in post-SCI inflammatory responses, we assessed glial reactivity using GFAP immunostaining. The siRNA@LNPs + GM-3Dpro group presented scar-free tissue repair without glial barrier formation at injury site. Furthermore, this group induced extensive parallel projection of 5-HT^+^ and NF200^+^ axons. Meanwhile, quantification data revealed that densities of 5-HT^+^ and NF200^+^ neural fibers in the siRNA@LNPs + GM-RA4IV and siRNA@LNPs + GM-3Dpro groups were significantly higher than that of the GM-RA4IV condition (Fig. 7E and F). Collectively, these results indicated that the GM-3Dpro scaffold with PTEN knockdown not only promoted robust parallel axon regeneration but also exhibited excellent biocompatibility and low immunogenicity. Furthermore, the presence of myelin and synaptic vesicles at the injury site further supported its great potential for enhancing transmission of neural signals.

### Molecular mechanism of parallel axon regeneration

**Fig. 8.**
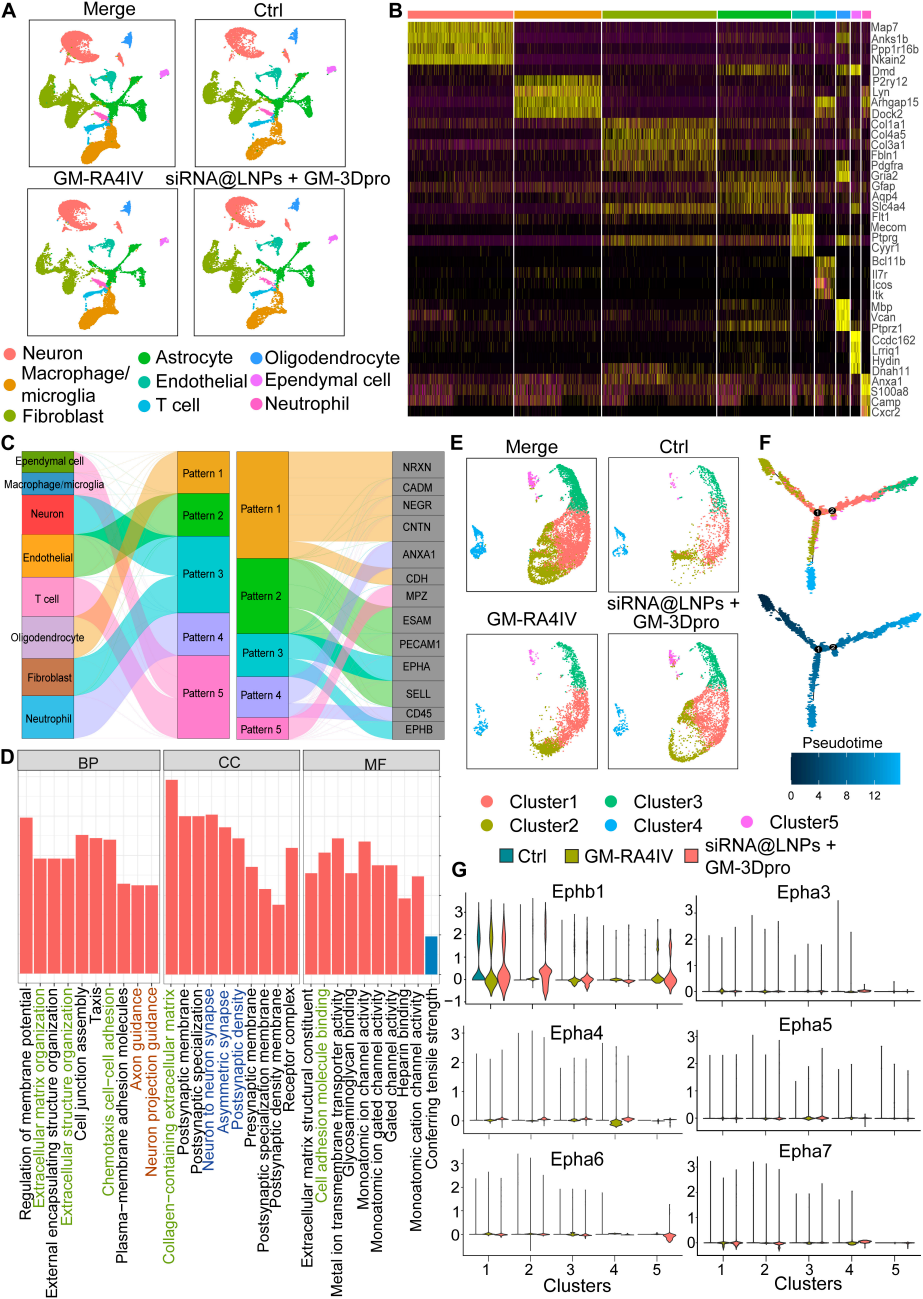
SC-RNA-seq analyses revealed mechanism of parallel orientation of regenerated axons in the siRNA@LNPs + GM-3Dpro group. (A) UMAP visualization of all cell types combined from Ctrl, GM-RA4IV, and siRNA@LNPs + GM-3Dpro based on DEGs. (B) Heatmap presented DEGs in different cell types. (C) Cell−cell connection between 9 cell types corresponding to different pathways and divided 5 patterns presented by river plotting. (D) GO enrichment by DEGs between Ctrl and siRNA@LNPs + GM-3Dpro groups at BP, CC, and MF. (E) UMAP visualization of 5 subclusters in neuron based on DEGs. (F) Cell differentiation trajectory in neuron subpopulation determined by pseudotime analysis (upper: differentiation trajectory of neuron subpopulations; down: differentiation trajectory presented by pseudotime). Circle number 1 and 2 indicated bifurcation of differentiation trajectory. (G) Expressions of Ephb1 and Epha3 to Epha7 in different neuron subpopulations.

To investigate the molecular mechanism of the siRNA@LNPs + GM-3Dpro treatment supporting parallel axon regeneration, we sought to examine gene expression levels through single-cell RNA sequencing (SC-RNA-seq). Firstly, 9 distinct cell clusters were identified in the spinal cord samples presented by uniform manifold approximation and projection (UMAP), where the proportion of neurons in the siRNA@LNPs + GM-3Dpro group (27%) was higher than that in the Ctrl (20%) and GM-RA4IV (22%) groups (Fig. 8A). Secondly, markers of each cluster were summarized in a heatmap, and representative marker genes expressed from different cell types were analyzed (Fig. 8B and Fig. S8). Among these cells, the ratio of immune cells, including macrophage/microglia, T cells, and neutrophils, was decreased in siRNA@LNPs + GM-3Dpro and GM-RA4IV conditions than in the Ctrl group (Fig. S9A). Meanwhile, the number of oligodendrocytes (a marker for mature axons) increased under the siRNA@LNPs + GM-3Dpro group. In addition, analysis of cell–cell communication revealed that the EPHA and EPHB pathways, which contributed to axonal guidance, were highly expressed in neurons and fibroblasts in the siRNA@LNPs + GM-3Dpro group (Fig. 8C).

Screening of DEGs among the 3 conditions followed by GO enrichment analysis revealed a specific gene expression pattern in the siRNA@LNPs + GM-3Dpro group. Compared to the Ctrl group, genes associated with ECM formation (Adamtsl2, Ddr2, Col1a2, Smoc2) [54–56], cell adhesion (Cntn6, Cdh8, Cdh12) [57,58], axon guidance (Epha4, Epha6, Ephb1, Robo2, Epha3) [39], and neuron synapse (Il1rapl2, Fgf13, Syndig1, Gabrb2) [59] were significantly enriched in the siRNA@LNPs + GM-3Dpro group (Fig. 8D). Further analysis of neuron cells revealed the presence of 5 distinct neuronal clusters (clusters 1 to 5; Fig. 8E). Among them, cluster 2 expressed genes related to neuron development such as Smarca1, Ppp1r1c, Tox, Ncald, Rfx4, and Cdh1, being a main contributor to the axon regrowth [60,61]. Pseudotime analysis figured out that cluster 2 existed at the origin of differentiation process, suggesting that the differentiation started with an activation of these cells (Fig. 8F). New neurons, including clusters 3, 4, and 5, were generated in the differentiation progression. Cluster 2 was differentiated into cluster 4, while cluster 2 was also differentiated into clusters 3 and 5 in a different direction. From quantification of different cell clusters in 3 conditions, clusters 1 and 5 increased, while cluster 4 decreased, which indicated an enhanced differentiation toward cluster 5 in the siRNA@LNPs + GM-3Dpro condition (Fig. S9B). As further determined by GO enrichment, clusters 1, 4, and 5 were enriched in genes related to synapse formation, locomotion recovery, cell–cell adhesion, and cell–ECM connection (Fig. S10). Therefore, the choice for differentiation toward cluster 4 or cluster 5 may be attributed to the axonal guidance function of the siRNA@LNPs + GM-3Dpro group. However, it was difficult to figure out the difference between clusters 4 and 5 through GO enrichment. Specifically, there were differences between the 2 neuron differentiation paths toward cluster 4 or cluster 5, and whether the siRNA@LNPs + GM-3Dpro group could improve axial guidance through the Ephrin/Eph pathway. Axonal guidance genes (Ephb1 and Epha3 to Epha7), mainly expressed in clusters 1, 2, and 5, were observed in the siRNA@LNPs + GM-3Dpro condition (Fig. 8G). Weighted gene coexpression network analysis revealed a gene expression network that included Ephb1 and Epha3 to Epha7, with Ephb1 connected to Epha3, Epha4, Epha5, and Epha7, suggesting a critical role for the Ephrin/Eph signaling pathway (Fig. S11). Combining the result that clusters 1 and 5 increased in the siRNA@LNPs + GM-3Dpro group, our analysis indicated that neuron cells in clusters 1, 2, and 5 were primarily responsible for axon regeneration. High expression of Ephb1 was confirmed by Western blotting. Subsequent immunostaining further showed that EphB1 colocalized with neurons in both the GM-RA4IV and siRNA@LNPs + GM-3Dpro conditions (Fig. S12). The siRNA@LNPs + GM-3Dpro group promoted axonal extension, improved adhesion of host neurons, and enhanced gene expressions involved in the Ephrin/Eph signaling pathway, which contributed to the parallel extension of regenerated axons.

## Discussion

The treatment of SCI is still a critical challenge as the lack of directed axonal growth and reconnection to appropriate targets have been major obstacles in achieving functional recovery. 3D-printed scaffolds alone provide only physical guidance for axonal regrowth but fail to address inhibitory molecular barriers or the hostile post-injury microenvironment. PTEN knockdown alone enhances intrinsic axonal regenerative capacity but does not provide structural support for directed regrowth or modulate the inhibitory extracellular milieu. Peptide hydrogels alone can optimize the extracellular microenvironment but lack the structural precision to guide long-distance axonal regrowth. Herein, we fabricated a triple synergy with a novel bioactive 3D-printed scaffold with parallel channels to significantly improve functional recovery of the hindlimb. Such scaffold not only protected spare tissue and prevented expansion of damaged area but also promoted axonal regeneration across the lesion site, effectively reconstructing the damaged neural circuitry. A key to the success of this strategy lay in the hydrogel design, which incorporated laminin-conjugated components that well mimicked the natural ECM fibrillar structure to provide essential regenerative signaling cues [28,62]. Therefore, the GM-RA4IV hydrogel modulated the immune response following SCI, orchestrated biological behavior of neural regeneration-associated cells, and guided regenerating axons along defined pathways by creating a permissive microenvironment that mimics the native ECM. Furthermore, preclinical studies have shown that conditional PTEN knockout in neurons reactivates mTOR signaling, leading to enhanced regenerative axon growth [8]. Thus, we successfully prepared the siRNA@LNPs and demonstrated that it significantly promoted axonal growth and extension by reducing the expression of PTEN protein based on extensive experiments in vitro and in vivo.

Immunohistochemical analysis revealed that the parallel channel structure of the hydrogel effectively induced parallel projection of regenerating axon tracts. Descending axons, including propriospinal axons and 5-HT axons originating from the cortex, were observed sprouting through the lesion site and aligning parallel to the natural axon structure. This finding was particularly significant, as long descending propriospinal axons receiving extensive descending inputs from the motor cortex were regarded as an important intraspinal coordination system for mediating reflex control and coordination during locomotion [63,64]. Furthermore, 5-HT axons that played a prominent role in locomotion have also been shown to promote functional recovery after SCI by increasing neurotransmission and altering motility of growth cones [42,43]. The multi-directional axons in the GM-RA4IV and siRNA@LNPs + GM-RA4IV groups were not functionally integrated into the neural circuitry, resulting in no significant improvement in locomotion under these conditions. Nevertheless, the combination of robust parallelly oriented long-distance axons created a complete neurite circuitry that contributed to functional recovery. Specifically, the parallelly oriented long-distance axons provided a path for descending neural signals from upstream, and meanwhile, the multi-directional axons integrated descending and ascending input to yield myokines [63,65].

SC-RNA-seq revealed a high expression of genes involved in the Ephrin/Eph signaling pathway that contributed to the parallel orientation of the regenerated axons despite the potential for deviation due to the larger diameter of the parallel channels (200 μm) compared to the axon fibers (2 to 3 μm). One of the unique features of the Ephrin/Eph pathway was the ability of Eph and Ephrin proteins to act as receptors and ligands simultaneously, leading to bidirectional or parallel and antiparallel signaling [38,63]. The SC-RNA-seq data highlighted the importance of specific Eph genes in the regeneration process. For example, neurons expressing Epha4 exerted an important function in synaptic transmission and led to cell proliferation [66]. Pseudotime analysis and the study of gene expression in neuron clusters indicated that the activation of the Ephrin/Eph pathway in the siRNA@LNPs + GM-3Dpro group depended on both high gene expression in clusters 1 and 2 and differentiation toward cluster 5 rather than cluster 4. Furthermore, the study shed light on the mechanisms by which the Ephrin/Eph signaling pathway established a precise organization of ascending and descending axonal tracts in the dorsal spinal cord. Repulsion and attraction functions were mediated by Ephrin-B:EphB and Ephrin-A:EphA signaling, respectively (Fig. S13), which were essential for the formation of 2 closely apposed axonal tracks that facilitated communication between the brain and spinal cord [39,67]. The role of Ephb1 expression in axon repulsion from the midline and ipsilateral trajectory projection was another significant finding of this study [68]. An elevated expression of Ephb1 in the regenerating axons within the parallel channel hydrogel suggested that this gene was actively involved in guiding axons along the desired ipsilateral trajectories, thus contributing to establishment of a functional axonal network. The concept of an axonal “highway” and “path” network, which encompassed both longitudinal dimensions and projection of axons along ipsilateral trajectories, was a novel and exciting aspect of this study. The “highway” indicates that longitudinal arranged axons transmit signals from brain to downstream, and the “path” indicates ipsilateral trajectories that play a role in transmitting signal to tissue; the combination of “highway” and “path” provides a complete system for neurite circles. A high expression of Eph genes in the regenerating axons, as revealed by the SC-RNA-seq, supported the formation of this network and highlighted the potential of such bioactive parallel channel hydrogel in promoting organized axonal regeneration.

In summary, we successfully prepared a multichannel 3D-printed scaffold integrated with laminin-derived peptide hydrogel and siRNA delivery system to facilitate effective axonal regeneration and motor functional recovery in a 3-mm T10 hemisection model in rats. The GM-RA4IV hydrogel created a favorable microenvironment for neural repair by modulating the immune response, providing neurotrophic support, and restricting lesion expansion. Meanwhile, the siRNA delivery activated intrinsic axonal regeneration ability by effectively knocking out the PTEN gene. Furthermore, parallel channel structure constructed by 3D printing provided crucial topological clues for directional growth of axons. Such synergistic strategy combining an activation of intrinsic regenerative potential with extrinsic physical guidance not only enabled effective axonal regeneration but also achieved precise axonal projection through activation of the Ephrin/Eph signaling pathway. Overall, this approach represented a highly promising novel pathway for SCI therapy and neural tissue engineering.

Further research is needed to optimize several significant barriers before clinical translation. First, the timing and method of intervention for SCI require further optimization. Although our approach was applied in an acute injury setting, its efficacy in chronic SCI, which represents the majority of clinical cases, remains to be determined. Second, the functionality and preliminary toxicology of siRNA and peptide were assessed in DRG cultures. This model is widely accepted in SCI research due to its robust axonal growth and practical utility. However, further validation using CNS neuron models is essential, as they more accurately mimic the native spinal cord environment. It is important to note that this study utilized a clinically relevant T10 hemisection model in rats, as a significant proportion of human SCIs are incomplete. The hemisection paradigm preserves the advantage of spared neural tissue and endogenous repair mechanisms, thereby providing a pathologically relevant context that more accurately simulates clinical scenario. Importantly, hemisection and complete transection models share critical similarities in their underlying cellular and molecular pathologies, including pronounced neuroinflammation, glial scar formation, and the up-regulation of inhibitory molecules within the lesion microenvironment. Our therapeutic strategy was designed to target these universal barriers to regeneration following SCI, providing a strong theoretical basis for its potential applicability in more severe injury models. However, we acknowledge that a complete transection model, by severing all innate axonal pathways, present a distinct and greater challenge. It not only eliminates the possibility of spontaneous recovery through spared tissue but also complicates scaffold-mediated regeneration by bypassing endogenous repair pathways. Furthermore, it more accurately recapitulates the most severe clinical cases characterized by large cavitary lesions and a profoundly inhibitory microenvironment. Therefore, while our findings in the hemisection model are promising, we agree that the critical next step is to evaluate the efficacy of our combinatorial approach in a complete transection model. Subsequent studies should also focus on validating this combinatory approach in complete transection models and in large animals, where additional challenges such as scaling, surgical delivery, and long-term implant stability must be thoroughly addressed.

## Materials and Methods

### Materials

Gelatin (type A, from porcine skin), methacrylic anhydride, lithium phenyl-2,4,6-trimethylbenzoylphosphinate (LAP), and PLL were purchased from Sigma-Aldrich Co. Ltd. (MO, USA). RA4IV peptide was obtained from BankPeptide (Hefei, China). PEGDA (1,000 Da), 1-(3-dimethylaminopropyl)-3-ethylcarbodiimide hydrochloride, N-hydroxysuccinimide, and monodisperse fluorescent microspheres (λex = 488 nm, λem = 518 nm, *d* = 2 μm) were purchased from Aladdin Biochemical Technology Co. Ltd. (Shanghai, China). Polyvinylidene difluoride membrane was purchased from Millipore (Bedford, MA, USA). Chicken anti-GFAP (ab134436, 1:500), rabbit NF200 heavy polypeptide (ab8135, 1:500), and rabbit anti-Tuj1 (ab18207, 1:500) were obtained from Abcam (Cambridge, UK). Rabbit anti-Ephb1 (AER-021,1:500) was purchased from Alomone Labs (Jerusalem, Israel). Mouse anti-NeuN (66836-1, 1:500) was purchased from Proteintech (Wuhan, China). Goat anti-chicken IgY (H+L) secondary antibody conjugated with Alexa Fluor 488, donkey anti-rabbit IgG (H+L) highly cross-adsorbed secondary antibody conjugated with Alexa Fluor 555, and donkey anti-mouse IgG secondary antibody conjugated with Alexa Fluor 647 were purchased from Invitrogen (Carlsbad, CA, USA). PTEN was synthesized by Tsingke Biotech Co. (Beijing, China). Donkey anti-rabbit secondary antibody conjugated with Alexa Fluor 488, rabbit anti-PTEN, rabbit anti-glyceraldehyde-3-phosphate dehydrogenase (GAPDH), and donkey anti-rabbit secondary antibody conjugated with horseradish peroxidase (HRP) were purchased from Abclonal (Beijing, China). Neurobasal medium, l-glutamine, and B27 were purchased from Gibco (Gaithersburg, MD, USA). Type I collagenase, pancreatin, streptomycin, and penicillin were purchased from Biological Industries (Shanghai, China). AAV2/9-syn-mCherry for axon tracing, siRNA for down-regulating, CCK-8, and Calcein/propidium iodide testing kit were purchased from Beyotime Biotechnology (Shanghai, China). 1,2-Dilinoleyloxy-3-dimethylamino-propane, 1,2-distearoyl-sn-glycero-3-PC (DSPC), cholesterol, and 1,2-dimyristoyl-rac-glycero-3-methoxypolyethylene glycol (DMG-PEG) were purchased from MedChemExpress (Monmouth Junction, NJ, USA).

### Synthesis of GM prepolymer

GM prepolymer was prepared according to a modified previously reported method [69]. Initially, 10.0 g of gelatin was dissolved in 100.0 ml of phosphate-buffered saline (PBS) at 50 °C with continuous stirring until homogeneous. Methacrylic anhydride (8.0 ml) was added dropwise to the above solution, and the mixture was allowed to react for 2 h at 50 °C. Subsequently, the solution was diluted with 100.0 ml of warm PBS and dialyzed against distilled water for 5 d using dialysis bags (12 to 14 kDa molecular weight cutoff). Finally, the GM prepolymer was collected after freeze-drying and stored at −20 °C for subsequent use.

### Syntheses of GM-PEGDA and GM-RA4IV hydrogels

For synthesis of the GM-PEGDA hydrogel, 10.0% (w/v) GM and 10.0% (w/v) PEGDA solutions were prepared in double-distilled water at 40 °C. The GM solution was then mixed with the PEGDA solution, and an equal volume of LAP was added to the mixture with a concentration of 0.25% (w/v). Finally, the above solution was exposed to 405-nm UV light for 10 s to form the GM-PEGDA hydrogel. For the synthesis of the GM-RA4IV hydrogel, EDC/NHS with a concentration of 12.0 mg ml^−1^ was added to 10.0% (w/v) GM prepolymer solution and stirred for 30 min to activate carboxyl groups. Then, to complete the reaction between carboxyl and amino groups, 20.0 mg of RA4IV peptide dissolved in 1.0 ml PBS was added to the above solution. Thus, the final mass fraction of RADA_4_-IKVAV in the GM-RA4IV hydrogel was approximately 2.0% (w/w). After adding LAP at a concentration of 17.0 mM, the GM-RA4IV hydrogel was constructed under 405-nm UV light for 10 s.

### 3D printing of GM-3Dpro scaffold

3D printing was performed according to our previous method [69]. Initial solutions containing 10.0% (w/v) GM, 10.0% (w/v) PEGDA, and 0.25% (w/v) LAP were sterilized using a 0.22-μm membrane filter. Digital 3D models were saved as STereoLithography files. The 3D-printed GM-PEGDA scaffold was fabricated with a highly defined parallel channel architecture to provide topographical guidance for axonal regeneration. The scaffold comprised 60 layers each with a thickness of 50 μm, resulting in a total height of 3 mm. Within this structure, 20 uniform parallel channels were arranged per layer, each measuring 200 μm in diameter and extending vertically throughout the entire scaffold. The soft GM-RA4IV bioactive hydrogel was then infused into these microchannels to form the final composite graft designated GM-3Dpro.

### Preparation of siRNA delivery system

The siRNA delivery system was crafted using a microfluidic formulation methodology, building upon previous research [70]. A mixture of lipids including (6Z, 9Z, 28Z, 31Z)-heptatriacont-6,9,28,31-tetraene-19-yl-4-(dimethylamino) butanoate (Dlin-MC3-DMA), DSPC, cholesterol, and DMG-PEG in a specific molar ratio of 50:10:38.5:1.5 was dissolved in ethanol, with an ionizable lipid concentration set to 2.0 mg ml^−1^. Meanwhile, siRNA stock solution with a concentration of 20.0 μg ml^−1^ was prepared in 10.0 mM citrate buffer at pH 4.0. These 2 solutions were rapidly combined to obtain the siRNA@LNPs using a microfluidic device, maintaining a total flow rate of 1.0 ml min^−1^ and an aqueous-to-ethanol volume ratio of 6:1. Then, the siRNA@LNPs was dialyzed in 10.0 mM PBS at pH 7.4 for 6 h using a Float-A-Lyzer G2 Dialysis Device with a 3.5-kDa molecular weight cutoff. To improve stability of the siRNA@LNPs, ultrasound was used to redisperse them for further use. LNPs were prepared following the same procedure but without incorporation of siRNA.

### Morphological characterization

SEM was employed to investigate morphologies of the GM and GM-RA4IV hydrogels. First, the hydrogels were rapidly frozen in liquid nitrogen, followed by freeze-drying at −50 °C. Before observation, all the samples were coated with a thin gold layer using a sputter coater. SEM imaging was performed using a Nova Nano SEM 230 (FEI Company, Hillsboro, OR, USA) to observe porous morphology of the hydrogels. Average pore sizes were measured from SEM images using ImageJ software (Media Cybernetics, Rockville, MD, USA).

### Swelling and degradation

For an evaluation of swelling ratio, the GM and GM-RA4IV hydrogels (15.0 × 12.0 × 10.0 mm^3^) were immersed in PBS solution at 37 °C. Then, mass difference at different time points was recorded. The swelling percentage was calculated as Eq. (1):

$$\text{Swelling}\;(\%)=\frac{W_{t}-W_{0}}{W_{0}}\times 100\%$$ (1)

where *W*_0_ is the initial weight of the hydrogel and *W*_t_ is the swollen weight of the hydrogel at a time point of *t*.

To evaluate the degradation behaviors in vitro, the GM and GM-RA4IV hydrogels (15.0 × 12.0 × 10.0 mm^3^) were completely immersed in PBS solution and placed in a constant-temperature shaker (37 °C) at a speed of 100 r·min^−1^. At the set time points, the PBS solution was exchanged, and the GM and GM-RA4IV hydrogels were weighed after absorbing surface water with filter paper. The degradation rate of the samples was calculated using Eq. (2):

$$\text{Degradation}\;(\%)=(1-\frac{W_{0}-W_{t}}{W_{0}})\times 100\%$$ (2)

where *W*_0_ is the initial mass of the hydrogel and *W*_t_ is the mass of the residue at the predetermined time point of *t*.

For the degradation test in vivo, 20 μl of the GM or GM-RA4IV hydrogels containing fluorescence microsphere with an excitation spectrum of 480 nm was injected into the injury site post-hemisection. After anesthetizing the rats with isoflurane at different time points, fluorescence intensity was tested by small animal in vivo imaging (IVIS Lumina III, PerkinElmer, USA). Fluorescence intensity at the injection site over time was quantified and normalized by photons captured per unit time per unit area.

### Encapsulation ratio

The encapsulation ratio of the siRNA@LNPs was measured using a RiboGreen RNA Quantification Kit (Solarbio, Beijing, China). First, a concentration standard curve was generated by preparing serial dilutions of siRNA standard, each mixed with an equal volume of RiboGreen working solution. Fluorescence intensity was measured using a fluorescence microplate reader (Tecan Spark, Männedorf, Switzerland) at an excitation/emission wavelengths of 500/525 nm. Subsequently, the fluorescence intensities of siRNA and siRNA@LNP samples were measured under the same conditions. Concentrations corresponding to the measured fluorescence intensities were determined using the standard curve. Finally, the encapsulation ratio was calculated using Eq. (3):

$$\text{Encapsulation ratio}\;(\%)=(1-\frac{C_{f}}{C_{t}})\times 100\%$$ (3)

where *C*_f_ is the concentration of siRNA in the siRNA@LNPs sample and *C*_t_ is the concentration of pure siRNA.

### Immunofluorescence staining of DRG neurons and quantification of extended axons

DRG neurons (1.0 × 10^5^ cells per well) were plated in the PLL-, GM-PEGDA-, or GM-RA4IV-coated plates and cocultured for 24 h at 37 °C with 5% CO_2_ environment. Subsequently, the cells were fixed with 4% paraformaldehyde (PFA) for 10 min, washed 3 times with PBS, and then blocked with a solution containing 0.3% Triton X-100 and 5% bovine serum albumin (BSA) for 1 h at room temperature. The fixed cells were incubated overnight at 4 °C with primary antibody. After that, the cells were exposed to secondary antibody for 3 h at room temperature. Following another round of PBS washing, the samples were examined and photographed using a confocal microscope (FV3000, Olympus, Japan). Sholl analysis by ImageJ software (Media Cybernetics, Rockville, MD, USA) was used to quantify average length of neurite branch in single cells based on the obtained images.

### Histological staining

Eight weeks post-injury, all rats were anesthetized by administering 1.0% pentobarbital sodium (5 ml kg^−1^). After confirming anesthesia, the rats underwent aortic perfusion with 4% PFA in 0.1 M PBS (pH 7.2 to 7.4). Their spinal cords were then extracted and fixed overnight in 4% PFA at 4 °C. Following fixation, these tissues were dehydrated by sequential immersion in 15.0% and 30.0% sucrose solutions, before being embedded in optimal cutting temperature compound. Sagittal sections were prepared using a cryostat (CM1950, Leica, Germany) and mounted on SuperFrost Plus slides (Fisher Scientific, USA). The sections were rinsed in PBS for 10 min and blocked with a solution of 0.3% Triton-X and 5.0% donkey serum for 1 h at room temperature. They were then incubated overnight at 4 °C with primary antibody solutions of GFAP, CD68, Iba1, 5-HT, neurofilament protein (NF200), and MBP, followed by a 3-h incubation at room temperature with secondary antibody, after 3 times of PBS washing. The sections were examined and photographed using a confocal laser scanning microscope (FV3000, Olympus, Japan). Six sections per animal were used, with 5 to 6 animals included per group. To analyze axon density in the lesion areas quantitatively, we measured irregular area surrounding the hydrogel implant and counted the axons within it. Finally, we normalized the axon count to the measured area of density (number/mm^2^) to obtain quantification of axon density.

For H&E staining, the dehydration step of spinal cords was the same as the previous step, but they needed to be prepared into longitudinal sections (thickness of 12 μm). The samples were then stained with H&E, and images were acquired using an Olympus upright microscope (BX63, Tokyo, Japan). To quantify the volume of newly formed tissues, gray values from the H&E-stained tissue were further analyzed. Measurements were taken at 0.5-mm intervals along a 4-mm span both rostral and caudal to the baseline at the injury epicenter. Each animal provided 5 sections for analysis, with 5 to 6 rats included per group.

### Behavior evaluation and EMG recording

Assessment of hindlimb movement of rats among different experimental groups was conducted in an open-field setting using the BBB scale, with the Intact group achieving the maximum scores. Detailed analysis of hindlimb kinematics employed the Vicon motion capture system following established protocols. Anatomical landmarks on the hindlimb, including iliac crest, hip joint, knee joint, ankle, and toe, were meticulously identified for precise tracking. Animals were positioned in a clear runway, and their gait cycles were recorded using the high-speed Vicon capture system. To assess locomotor function, videos were thoroughly analyzed using Vicon Nexus software by measuring critical metrics such as iliac crest height and stride length based on identified landmarks. Additionally, EMG recordings were conducted on freely moving animals, following established procedures. Three rats per group (Intact, Ctrl, GM-PEGDA, GM-RA4IV, siRNA@LNPs + GM-3Dpro) were randomly selected at 8 weeks post-surgery. Bipolar electrodes (AS632, Conner Wire, UK) were surgically implanted into GS and TA. Electrode wires were subcutaneously routed along the rat’s back and anchored to the skull for ensuring stability. EMG signals were captured using a differential neuron signal amplifier (BTAM01L, Brain Tech, Nantong, China) in a 30- to 2,000-Hz filtration range, sampled at 30 kHz via the Neurostudio system (Brain Tech, Nantong, China). Finally, data analysis utilized custom MATLAB scripts to comprehensively assess muscle activity patterns, providing insights into locomotor function across experimental groups.

### Western blotting

Total proteins were extracted from 1.5-mm tissue segments of the spinal cord, harvested from regions immediately rostral and caudal to the injury site in rats. Specifically, the proteins were extracted using a lysis buffer and centrifuged at 12,000*g* for 10 min at 4 °C. Protein (50.0 mg) was separated on 10% polyacrylamide gel via sodium dodecyl sulfate–polyacrylamide gel electrophoresis and subsequently transferred onto polyvinylidene difluoride membranes. The membranes were blocked with 5% skimmed milk in PBS with Tween 20 (PBST) for 1 h at room temperature and then incubated overnight at 4 °C with the primary antibody. After washing with PBST, the membranes were incubated with the secondary antibody for 1 h at room temperature. Finally, protein bands were detected using an ECL kit and imaged with a ChemiDoc system (Bio-Rad, CA, USA).

For in vivo Western blotting detection experiment of nuclide delivery, the siRNA@LNPs were injected into the T10 segment of the rat spinal cord using a micropipette (RWD, Shenzhen, China). Briefly, a pulled-glass micropipette connected to a 10-μl Hamilton microsyringe was utilized. Then, an injection of siRNA@LNPs was performed at 3 different depths (0.6, 1.2, and 1.8 mm) in the exposed spinal cord at a rate of 200 nl min^−1^ at 4 sites (1 μl per site). Tissue samples from the injection sites with 2 mm were collected 2 weeks post-injection, and total proteins were extracted for subsequent analysis.

### RNA-seq analysis

Eight weeks after surgery, spinal cord tissue samples of rats were harvested from regions 1.5 mm rostral and caudal to the injury epicenter for RNA sequencing. Each experimental group included 3 animals. Total RNA extraction from the tissue samples was performed utilizing TRIzol reagent, followed by deoxyribonuclease treatment to eliminate genomic DNA contamination. Quality of the extracted RNA was rigorously assessed, and only RNA samples with an RNA integrity number value exceeding 8 were deemed acceptable for subsequent procedures. Subsequently, 1.0 μg of high-quality RNA was employed to construct libraries using the TrueSeq RNA Access Library Prep Kit (Illumina, CA, USA), in strict accordance with the manufacturer’s protocols. For the analysis, the rat genome sequence and corresponding annotations retrieved from the ENSEMBL genome database served as references. Differential gene expression analysis was conducted based on a model incorporating variance-mean dependence and adhering to the negative binomial distribution. To further explore the biological significance of DEGs, GO enrichment analysis was performed. The entire data analysis pipeline harnessed the power of R packages, including DESeq for differential expression analysis and specialized GO enrichment tools.

### SC-RNA-seq analysis

Animals were deeply anesthetized with 1% pentobarbital sodium (0.5 ml/100 g) 8 weeks post-injury, and then a 4-mm spinal cord segment centered on the injury site was excised and immediately frozen at −80 °C. The frozen samples were homogenized in ice-cold nuclei EZ lysis buffer using pestles A and B, followed by incubation. Nuclei were subsequently centrifuged, washed, and resuspended to achieve a concentration of 1,000 nuclei per μl. The suspension was loaded onto a Genomics Chromium Channel, along with beads containing unique molecular identifiers (UMIs) and cell barcodes. Polyadenylated RNA molecules were hybridized to the beads, and reverse transcription was performed to tag complementary DNA molecules at the 3′ end with UMIs and cell labels. The process continued with second-strand cDNA synthesis, adaptor ligation, and amplification. Sequencing libraries were prepared, quantified, and sequenced on the NovaSeq 6000 platform at Shanghai Majorbio Bio-pharm Technology Co. Ltd. For data analysis, sequencing reads were processed using Cell Ranger to generate a gene-barcode matrix. Quality control and filtering were carried out with Seurat, retaining cells that expressed 200 to 2,500 genes and had less than 5% mitochondrial reads. Subsequent steps included normalization, cell clustering, marker gene identification, and GO enrichment analysis, all performed using R packages.

### Statistical analysis

All quantitative data were obtained from at least 3 independent experiments and were expressed as the mean ± standard deviation (SD). To compare means among the 3 groups, one-way analysis of variance (ANOVA; GraphPad Prism, version 8.0, San Diego, CA, USA) was used, and a *t* test was applied for comparisons between 2 groups. Significance was defined as *P < 0.05, **P < 0.01, and ***P < 0.001 for all statistical tests. GraphPad Prism and Origin Pro (version 8.5, Northampton, MA, USA) were used for plotting and figure preparation.

## Data Availability

The data that support the findings of this study are available from the corresponding authors upon reasonable request.
