# Supplementary material for: Multichannel 3D-Printed Bioactive Scaffold Combined with Small Interfering RNA Delivery to Promote Neurological Recovery after Spinal Cord Injury
Source: Research (Wash D C). 2025 Oct 21;8:0951. doi: 10.34133/research.0951 (PMC12538118; doi:10.34133/research.0951)
Supplement: Supplementary 1 — Supplementary Methods Figs. S1 to S13 Tables S1 and S2 [file research.0951.f1.docx]

Supplementary Information

Multichannel 3D-printed bioactive scaffold combined with siRNA delivery system to promote neurological recovery after spinal cord injury

*Jingjia Ye^d,1^, Fenglu Li^b,c,1^, Zhengfa Wen^a^, Junsheng He^a^, Gaoxing Pan^b,c^, Xinrang Zhai^d^, Linran Song^a^, Xianzhu Zhang^e^, Xuefei Zhou^a^, Xudong Yao^a^, Yanlang Wang^f^, Jin Zhang^b,c^*, and Wei Wei^a^**

^a^ Center for Regeneration and Aging Medicine, the Fourth Affiliated Hospital of School of Medicine, and International School of Medicine, International Institutes of Medicine, Zhejiang University, Yiwu, Zhejiang, 322000, P. R. China

^b^ Qingyuan Innovation Laboratory, 1 Xueyuan Road, Quanzhou 362801, P. R. China

^c^ College of Chemical Engineering, Fuzhou University, 2 Xueyuan Road, Fuzhou 350108, P. R. China

^d^ School of Chemistry and Chemical Engineering, Nanjing University of Science and Technology, Nanjing, Jiangsu, 210094, P. R. China

^e^ Department of Sports Medicine of the The First Affiliated Hospital, and Liangzhu Laboratory, Zhejiang University School of Medicine, Hangzhou 310003, P. R. China

^f^ School of Advanced Manufacturing, Fuzhou University, 1 Shuicheng Road, Jinjiang 362200, P. R. China

* Corresponding authors.

E-mail addresses for corresponding: [zjewwei@zju.edu.cn](mailto:zjewwei@zju.edu.cn) (W. Wei), [J_Zhang929@fzu.edu.cn](mailto:J_Zhang929@fzu.edu.cn) (J. Zhang).

^1^ J. Ye and F. Li contributed equally to this work.

**Supplementary** **Methods**

**1. Methods**

**1.1 Compression performance**

Compression properties of the gelatin methacryloyl (GM), gelatin methacryloyl-RADA4-IKVAV (GM-RA4IV), and gelatin methacryloyl-poly(ethylene glycol) diacrylate (GM-PEGDA) hydrogels were evaluated using a universal testing machine (C41, MTS, USA) at room temperature. Three cylindrical samples with a diameter of 10.0 mm and height of 2.0 mm were prepared for each type of hydrogel. All samples were compressed to obtained compressive stress−strain curves at a speed of 1mm min^−1^, and compressive modulus was calculated at a linear region of 10%−20% strain.

**1.2 R****heological analysis**

Rheological test of the GM-RA4IV hydrogel was performed using a rheometer (MCR302, Anton Paar, Austria). Specifically, the hydrogel precursor solution (0.4 mL) was injected between parallel steel plates with an adjustable distance of 500 μm and strain of 3%. Then, storage modulus (*G'*) and loss modulus (*G*″) of the solution were recorded by exposing it to UV light (30 mW cm^−2^, 405 nm) for 30 s. Additionally, time-scan analyses were performed at a fixed strain of 1% and an angular frequency of 1.0 Hz, to compare changes in *G'* and *G*″ of GM-RA4IV hydrogel with a diameter of 10.0 mm and height of 2.0 mm.

**1.3 Size and zeta potential**

The average particle sizes and zeta potentials of the liposome nanospheres (LNPs) and liposome nanospheres loaded with siRNA (siRNA@LNPs) were measured using dynamic light scattering and zeta potential analysis (Zetasizer Nano Series, Malvern Instruments Ltd., Malvern, UK). Specific testing steps taken the siRNA@LNPs as an example. For size measurement, 100 µL of siRNA@LNPs was dispersed in 10.0 mL of anhydrous ethanol. A 1.0 mL aliquot of this solution was analyzed for particle size at a wavelength of 633 nm with a scanning frequency of 30 cycles. Measurements of zeta potential were carried out at 25 ℃, with at least three cycles for each sample.

**1.4 Rat dorsal root ganglions (DRG) neurons isolation and cells culture**

DRG neurons represented a useful model to study axon regeneration were isolated and purified from lumbar of spinal column in day 0−1 postnatal Sprague-Dawley (SD) rats following an established method [1, 2]. Briefly, DRG tissues were collected in ice-cold phosphate buffer saline with 200 U mL^−1^ penicillin/streptomycin. Subsequently, the tissues dissected into small pieces were transferred to a sample tube containing 3.0 mL of type I collagenase (3.0 mg mL^−1^), and incubated for 90 min at 37 ℃ with 5% CO_2_ environment. After an incubation, centrifugation was carried out for 5 min at 1,200 rcf to remove the collagenase, and the DRG pieces were then resuspended in 3.0 mL of pancreatin for 5 min. 10% fetal bovine serum was added to the above solution for halting the digestion process, which was subsequently filtered through 100 and 70 μm cell strainers. Finally, DRG neurons were collected by centrifugation (900 rcf, 5 min) and purified using 15% bovine serum albumin. For the cell culture, the purified DRG neurons were cultured in a neurobasal medium supplemented with 2% B27, 1% glutamine, and 1% streptomycin and penicillin.

**1.5 Biocompatibility assay**

Cell cytotoxicity of PLL-, GM-PEGDA-, and GM-RA4IV-coated plates was assessed using PC12 cells (Procell, Wuhan, China). PC12 (1.0 × 10^4^ cells/well) were cultured with different samples in 96-well plates at 37 ℃ under 5% CO_2_. Live/Dead staining test was performed according to the manual of the Calein/PI testing kit at 24 h. To assess cell viability, CCK-8 test was further performed based on the manual. Briefly, DRG neurons (1.0 × 10^4^ cells/well) were *co*-cultured with the samples (PLL-, GM-PEGDA-, and GM-RA4IV-coated plates) for 24 h. Then, 100.0 μL of 10% (*V/V*) CCK-8 solution was added to each culture plate, and the cells were incubated at 37 ℃ with 5% CO_2_ for 3 h away from light. Finally, 100.0 μL of culture medium was collected from each well, and optical density values were measured at 450 nm using a microplate reader (Thermo Fisher, MA, USA). All the samples used in cell experiments were disinfected by washing twice in 75% ethanol and irradiating with ultraviolet light for 30 minutes in sequence. In addition, the LNPs and siRNA@LNPs with a concentration of 10.0 μL mL^−1^ were added to the neural substrate culture medium and *co*-cultured with DRG neurons (1.0 × 10^4^ cells/well) for 24 h. The steps of CCK-8 detection were repeated to verify the level of cell apoptosis.

**1.6 Quantitative real-time polymerase chain reaction (****RT-PCR) assay**

A siRNA sequence targeting GGCTAAGTGAAGACGACAA was designed, with a sense strand being *5'*-GGCUAAGUGAAGACGACAA-*3'* and an antisense strand being *5'*-UUGUCGUCUUCACUUAGCC-*3'*. To improve stability *in vivo*, the siRNA was modified with Fam at the *5'* end and included a *2*-methoxy modification. The prepared NPs and siRNA@LNPs (10.0 μL mL^−1^) were added to the neurobasal medium and *co*-cultured with DRG cells (1.0 × 10^4^ cells/well) for 48 h. Total RNAs from DRG neurons using RNeasy Plus Mini Kit (TAKARA, Dalian, China) with a Biorad CFX96 PCR system (Biorad, Astralia). Relative RNA expression levels were calculated by RT-PCR and normalized to GAPDH. All PCR amplifications were performed in 20.0 µL of final reaction mixture, and the relative primer sequences were listed in Table S1. Relative expressions of target genes were calculated using the 2^−ΔΔCt^ method.

**1.7 Surgery for hemisection spinal cord Injury (SCI) and scaffold grafting**

Thirty adult female SD rats weighing 250 ± 10 g were procured from the Zhejiang Academy of Medical Sciences. Zhejiang University Animal Experimentation Committee granted approval for all research procedures, ensuring strict adherence to the guidelines set forth by the National Institutes of Health Guide for the Care and Use of Laboratory Animals (ZJU20220394). These SD rats were randomly divided into six groups: Intact, Ctrl, GM-PEGDA, GM-RA4IV, and siRNA@LNPs + GM-PEGDA/GM-RA4IV (siRNA@LNPs + GM-3Dpro). Construction of a spinal cord hemisection injury model adopted the method previously reported [3]. In brief, rats were anesthetized *via* intraperitoneal injection of 1% pentobarbital sodium (5 mL kg⁻^1^). Once anesthetized, they were secured in a prone position on a surgical table. A laminectomy was performed to expose the T10 spinal cord, after which a segment approximately 3 mm in length was excised unilaterally using an iris knife to create a cavity. Semi-cylindrical scaffolds measuring 2 mm in radius and 3 mm in length were then precisely implanted into the defect. Post-surgery, the muscle and skin layers were sutured closed, and manual bladder expression was performed twice daily until the end of the experimental period. Six weeks post-injury, anterograde tracing of regenerated axons was carried out by injecting AAV2/9-mCherry into the T7–8 spinal segment. Specifically, the viral vector was delivered using a glass micropipette at a rate of 100 nL min⁻^1^. Injections were made 0.4 and 0.8 mm lateral to the midline, rostral to the injury site. A total volume of 150 nL was administered per injection site, distributed across three depths: 0.6, 1.2, and 1.8 mm below the dorsal surface. All rats were sacrificed for analysis at eight weeks post-injury. All conditions for animal experiments are described in Table S2 (Supporting Information).

**Supplementary Figures**


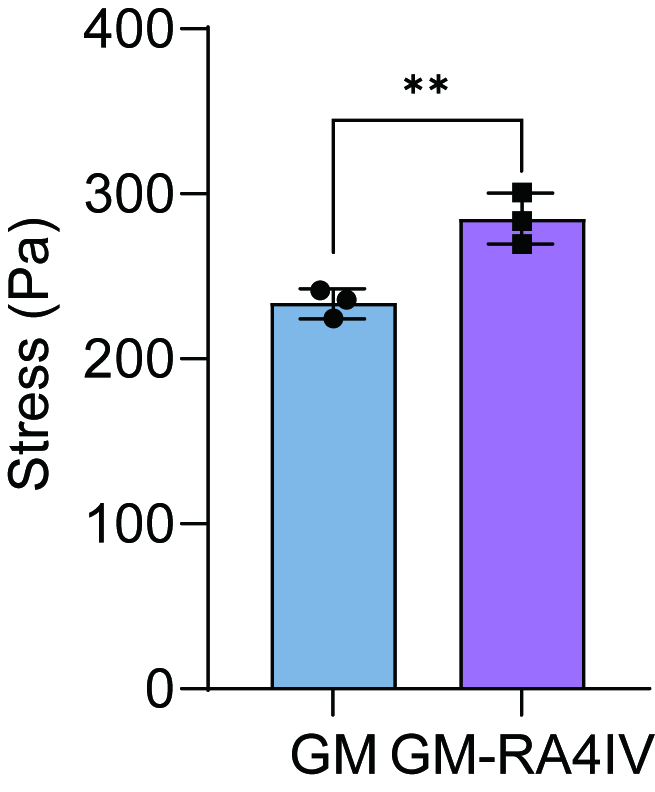


**Fig. S1.** Compressive strengths of GM and GM-RA4IV hydrogels (*n* = 3). All statistical data are represented as mean ± SD (***P* < 0.01). T-test was used.


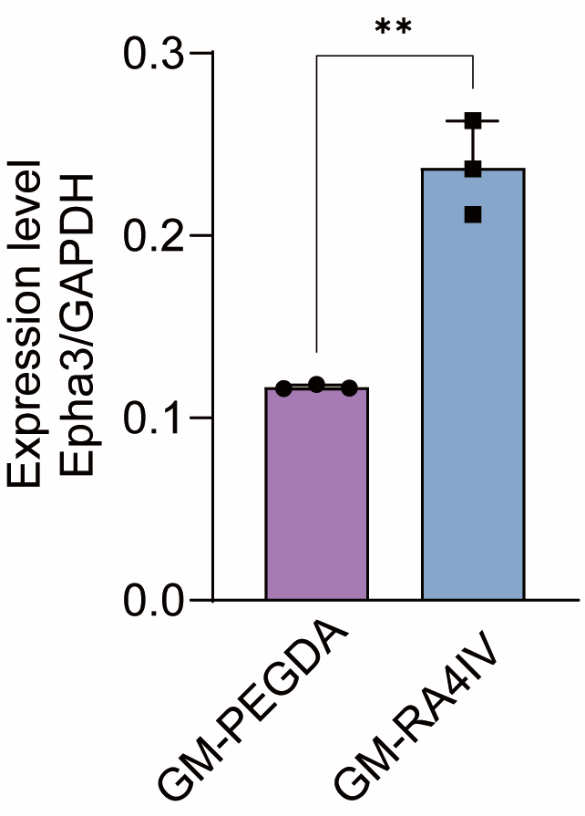


**Fig. S2.** Gene expression levels of Epha3 of DRG cells *co*-cultured with GM-PEGDA- and GM-RA4IV-coating plates (*n* = 3). All statistical data are represented as mean ± SD (***P* < 0.01). T-test was used.

**
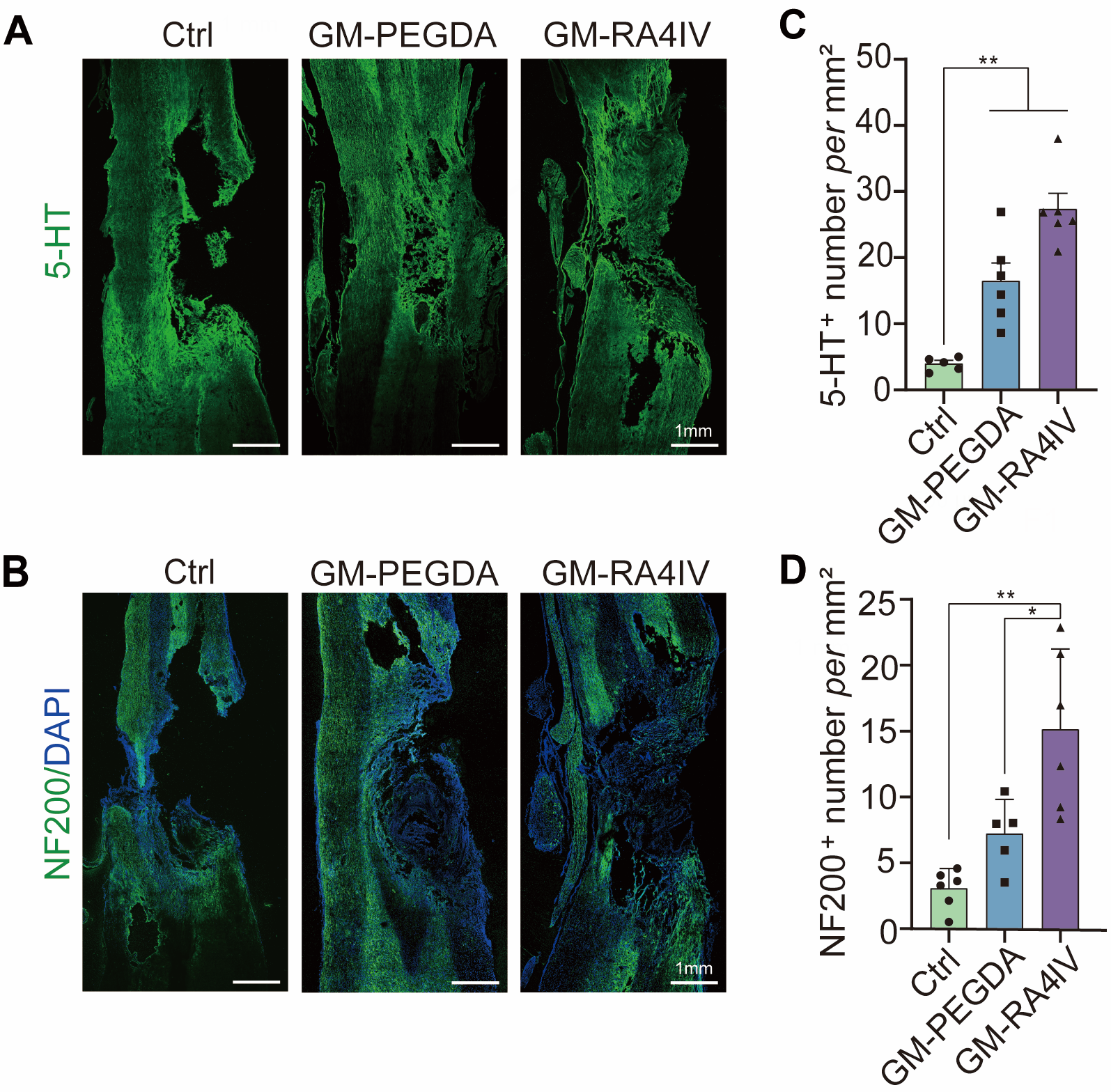
**

**Fig. S3.** **Immunochemistry of 5-HT and** **NF200 axonal fibers.** Immunochemistry images of (A) 5-HT and (B) NF200 axonal fibers at injury site in Ctrl, GM-PEGDA, and GM-RA4IV conditions. Quantitative analyses of (C) 5-HT^+^ and (D) NF200^+^ axons *per* mm^2^ based on immunochemistry images. (*n* = 6), All statistical data are represented as mean ± SD (**P* < 0. 05; ***P* < 0.01). One-way ANOVA followed by Tukey's test was used in (C and D).

**
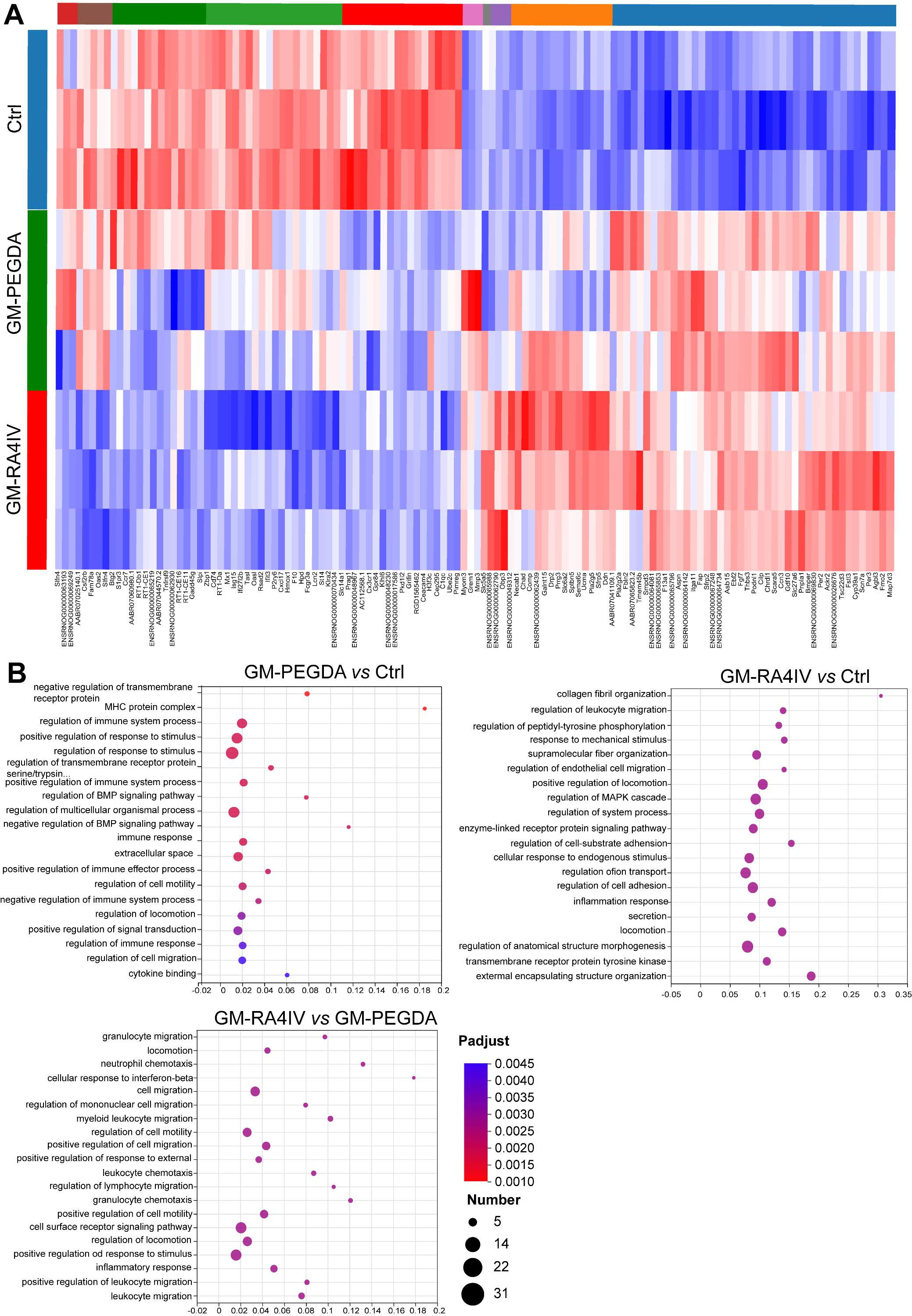
**

**Fig. S4. Effect of Gel-RA4IV in relieving immune reaction and improving locomotion function as revealed by RNA sequence.** (A) Heatmap presenting DEGs in Ctrl, Gel-PEGDA, and Gel-RA4IV groups. (B) GO enrichment of DEGs in 3 groups against each other.


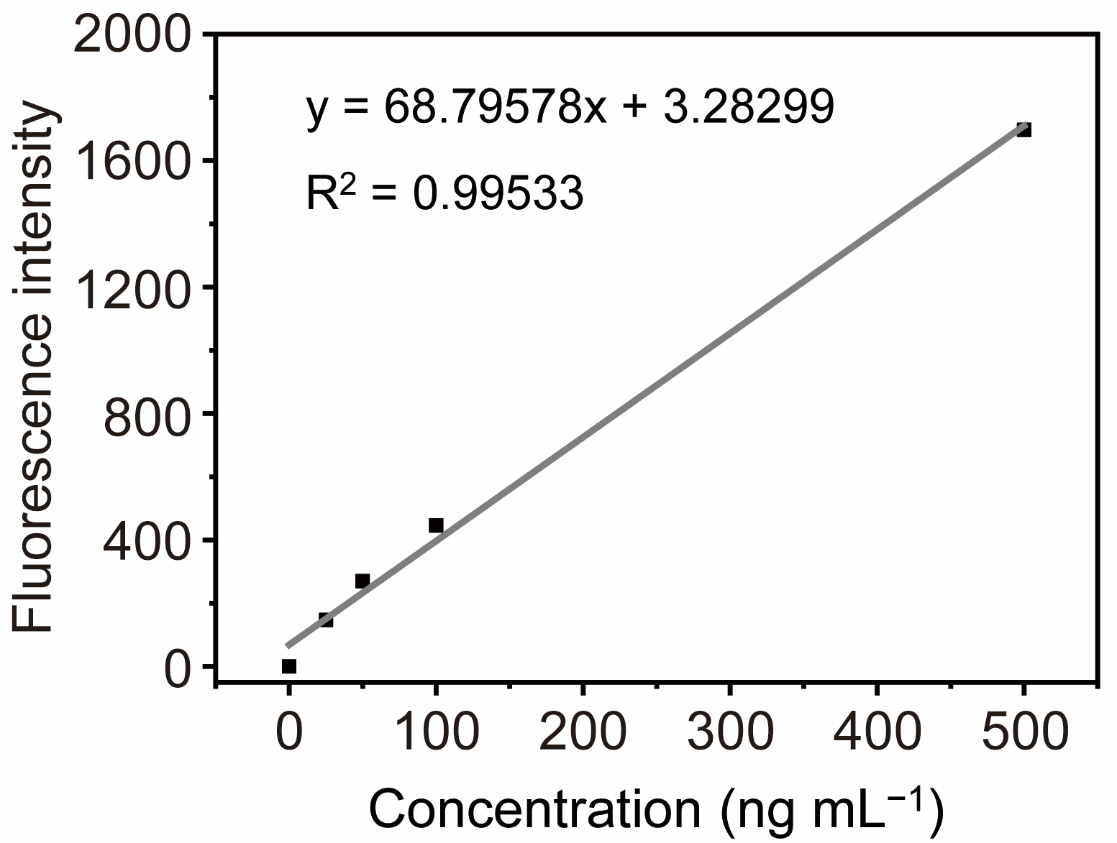


**Fig. S5.** A concentration standard curve of siRNA.


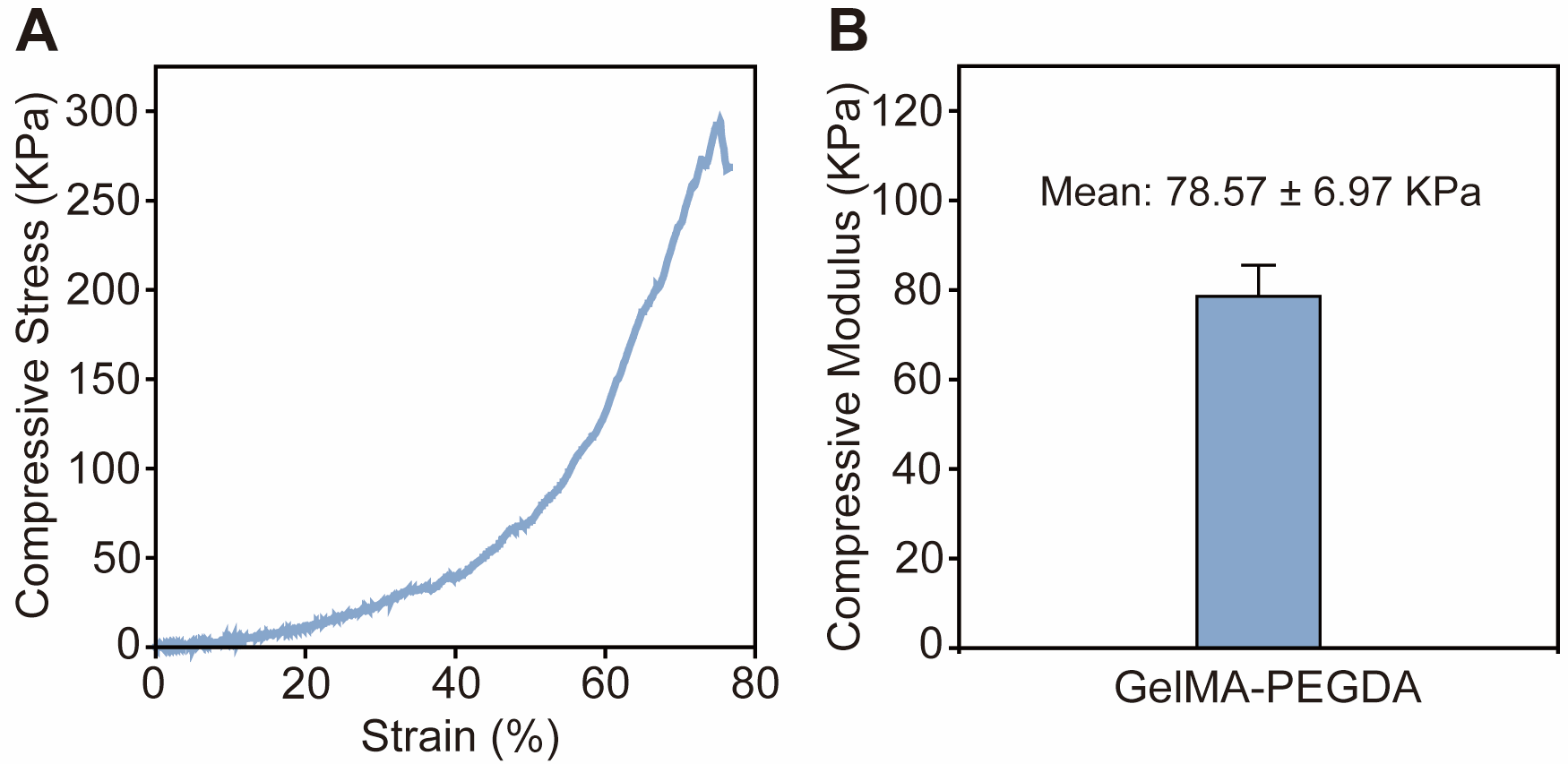


**Fig. S6. Mechanism properties of GM-PEGDA hydrogel.** (A) Compressive stress−stain curve and (B) modulus of GM-PEGDA hydrogel (*n* = 3). All statistical data are represented as mean ± SD.

**
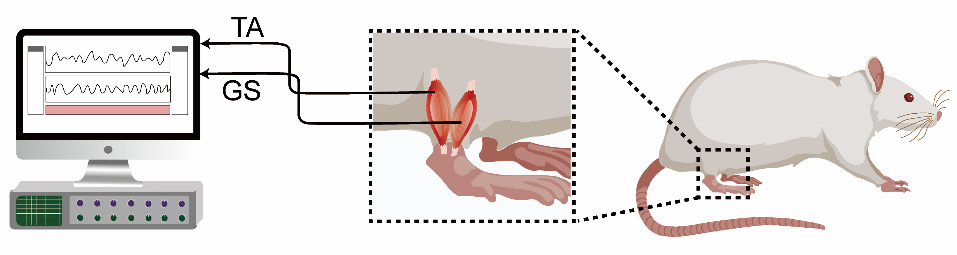
**

**Fig. S7** Schematic diagram of EMG recording.


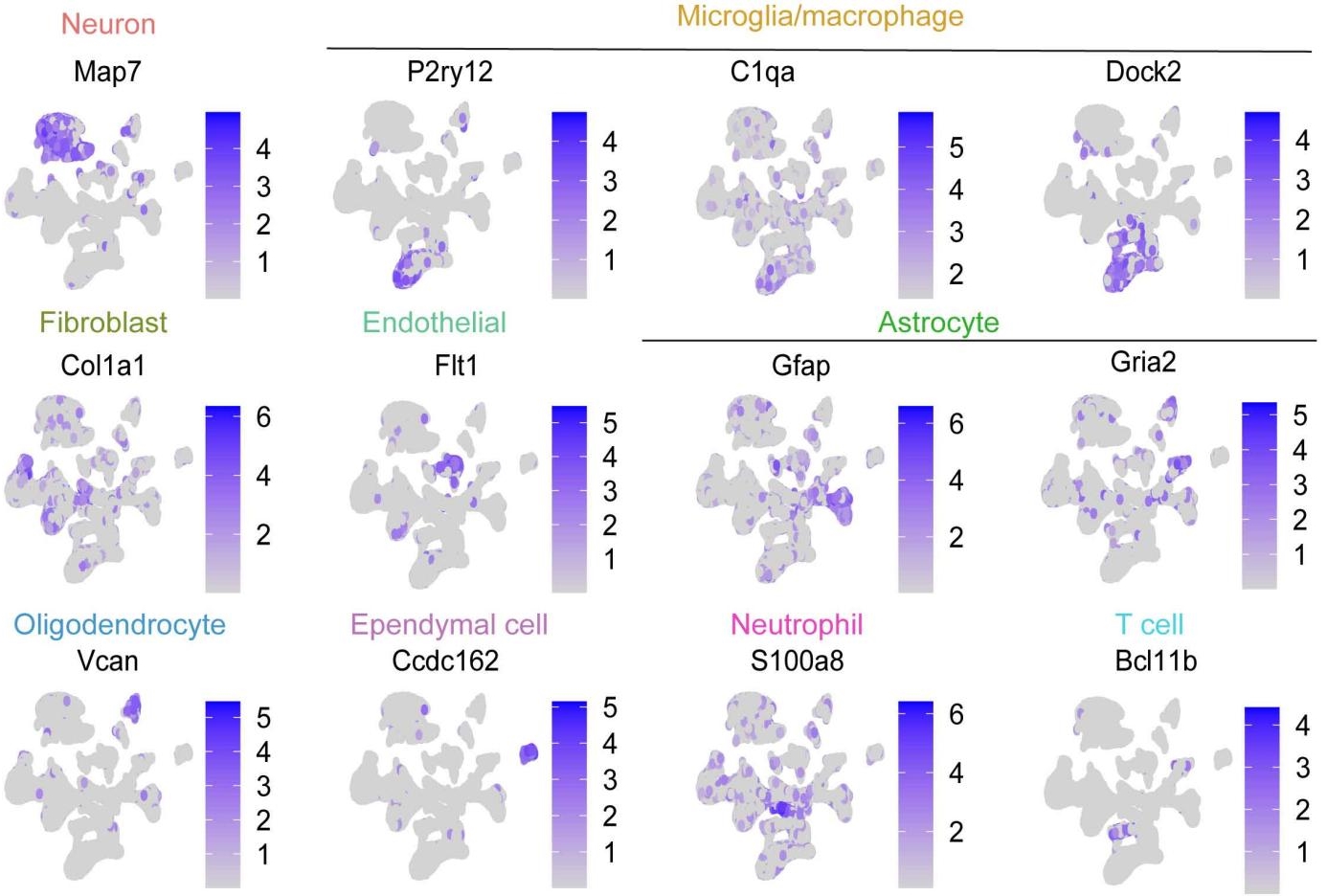


**Fig. S8.** Marker genes expressed from different cell types.

**
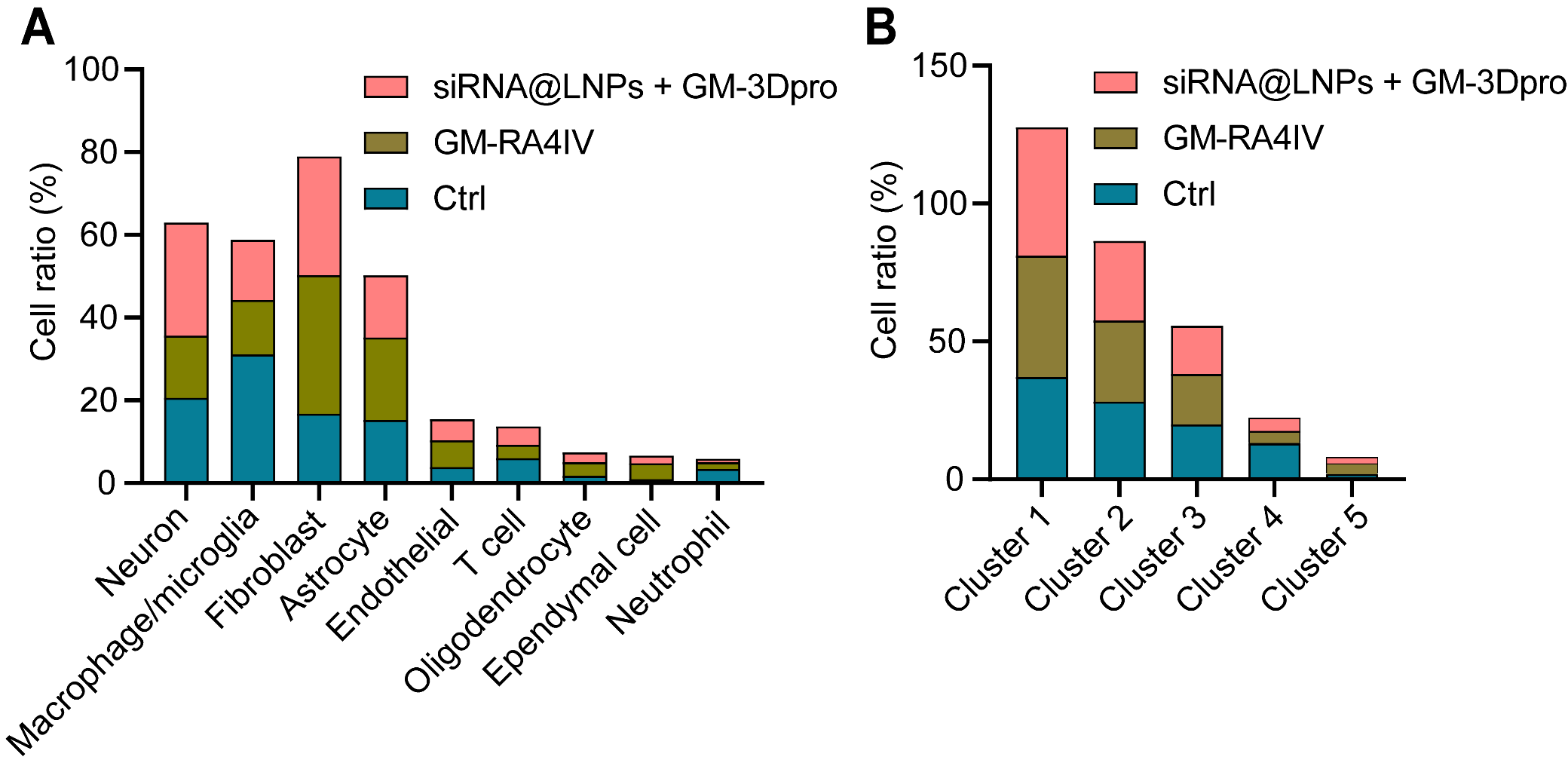
**

**Fig. S9. Cell ratios in different conditions.** Cell ratios of different (A) neuron subtypes and (B) culsters in Ctrl, GM-RA4IV, and siRNA@LNPs + GM-3Dpro conditions.

**
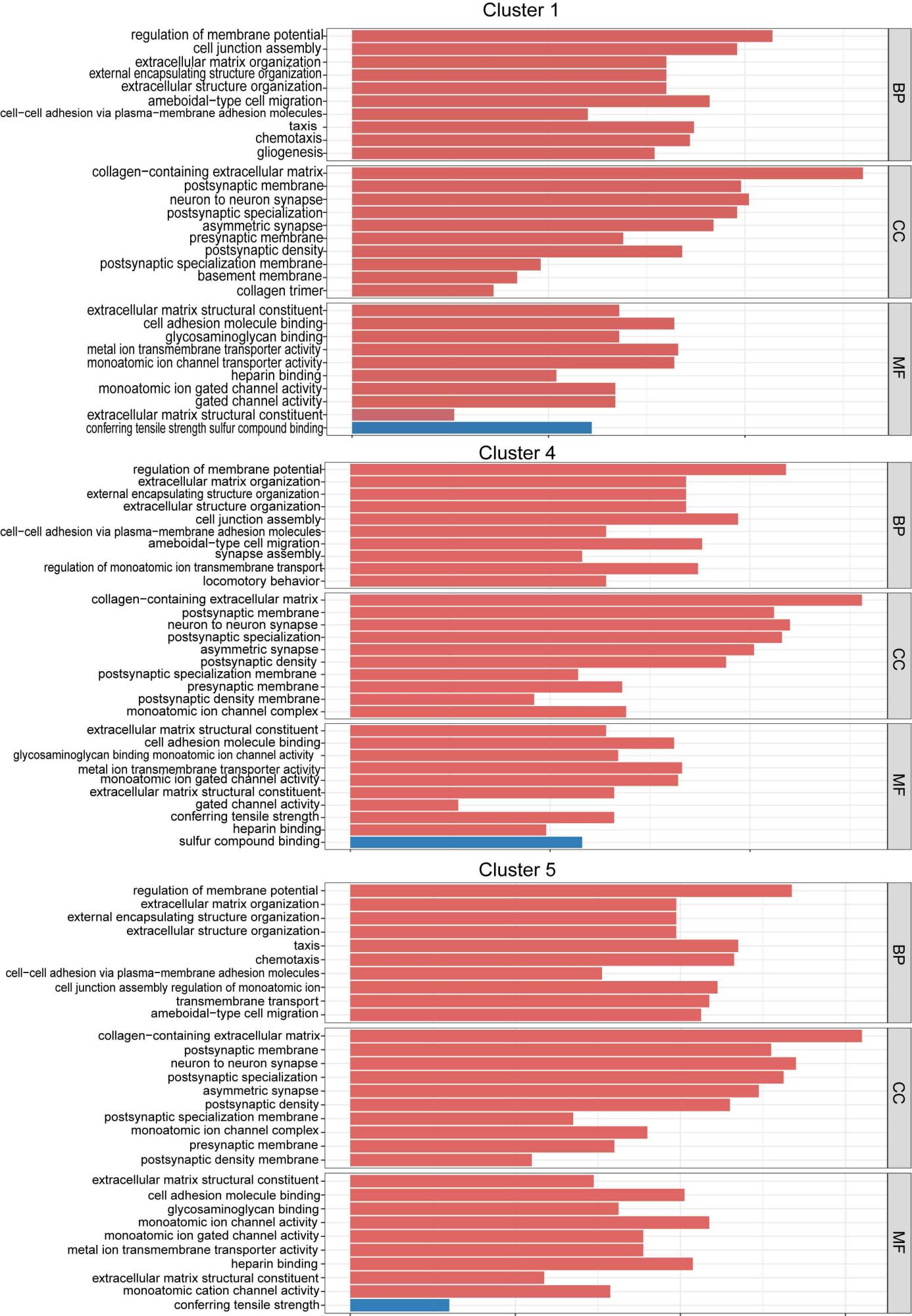
**

**Fig. S10.** GO enrichment of DEGs in neuron subpopulation cluster 1, 4 and 5.
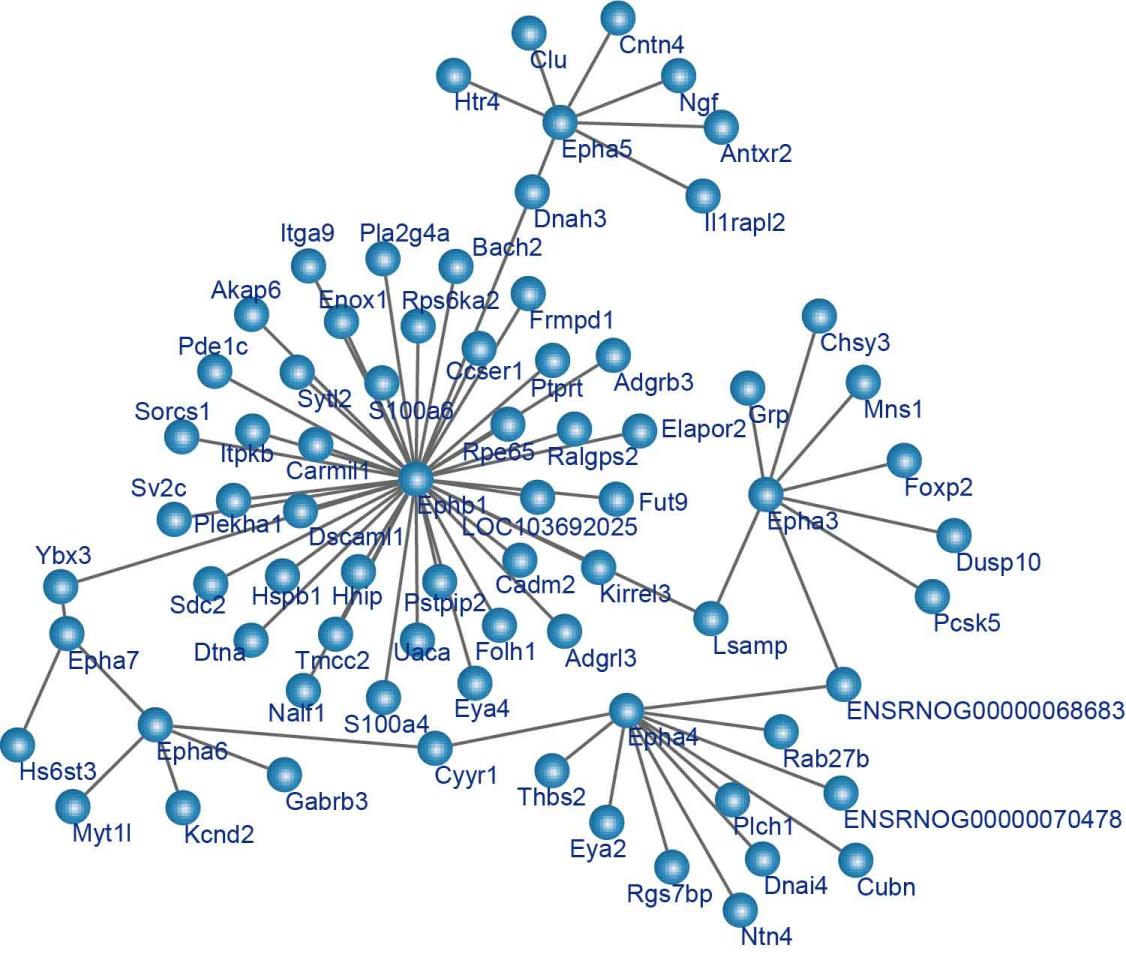


**Fig. S11.** Weighted correlation network analysis showing gene-expression network in siRNA@LNPs + GM-3Dpro group.

**
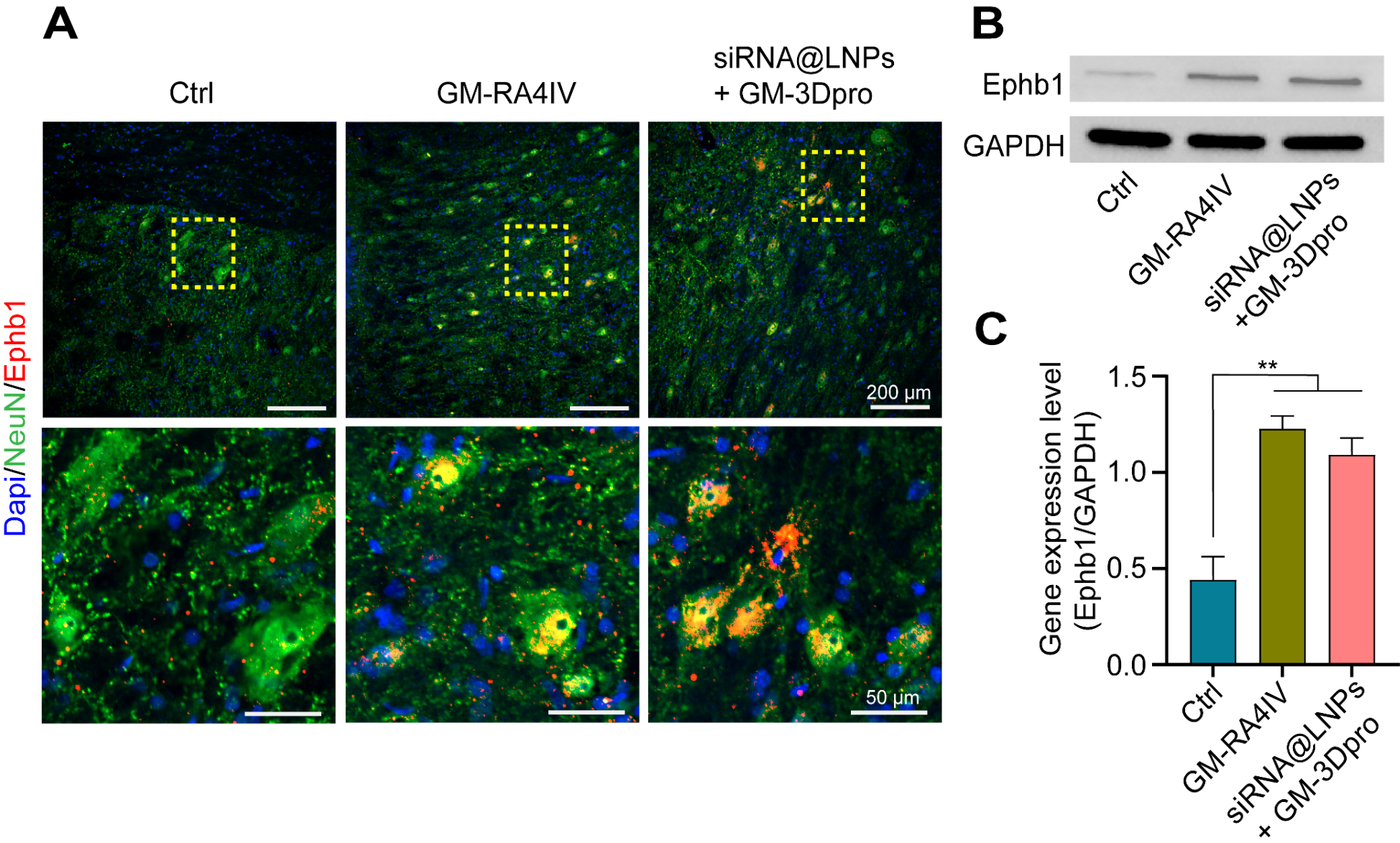
**

**Fig. S12.** **Improved gene expression of Ephb1 in GM-RA4IV and** **siRNA@LNPs + GM-3Dpro groups.** (A) Immunohistochemical staining images presenting Ephb1 and NeuN positive cells in Ctrl, GM-RA4IV, and siRNA@LNPs + GM-3Dpro groups. (B) Western blotting showing expression level of Ephb1 in three conditions. (C) Quantification of Ephb1 based on western blotting (*n* = 3). All statistical data are represented as mean ± SD (***P* < 0.01). One-way ANOVA followed by Tukey's test was used in C.

**
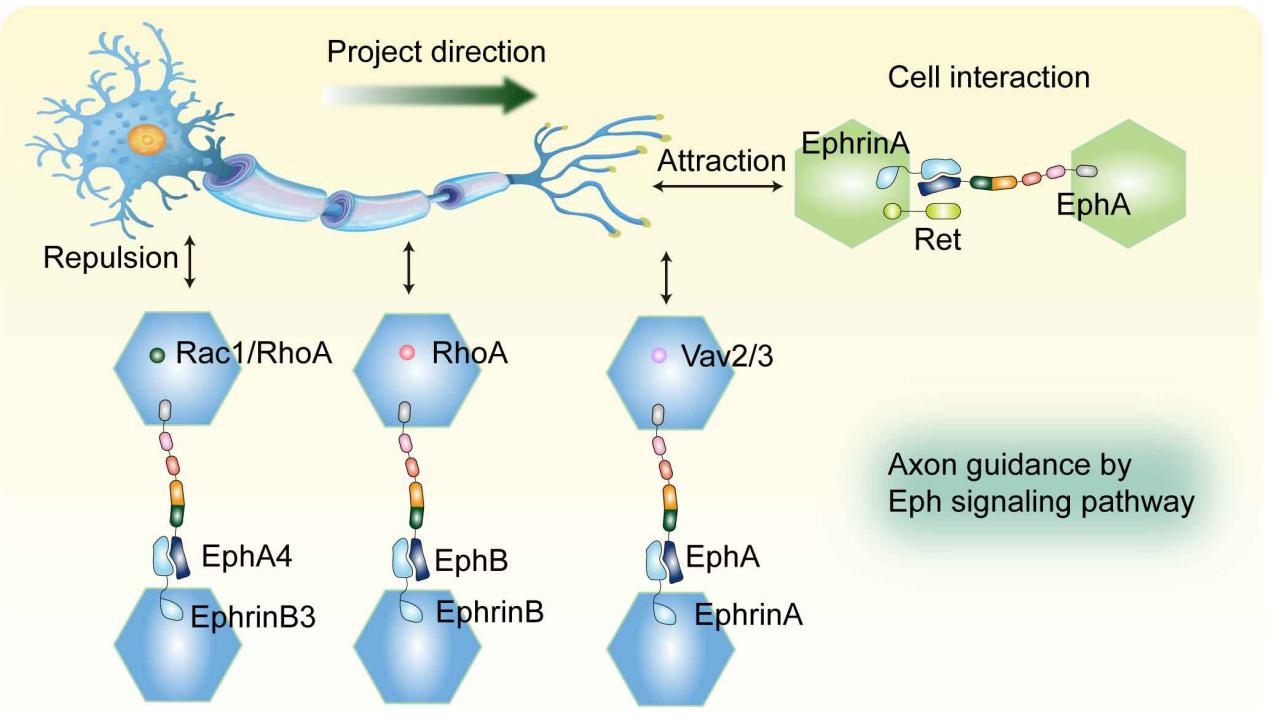
**

**Fig. S13.** Schematic diagram showing mechanism of Ephrin/Eph signaling pathway in axon guidance.

**Supplementary Tables**

**Table S1.** Forwards and reverse primer sequencies for RT-PCR

| Gene | Forwards | Reverse |
| --- | --- | --- |
| Ephb2 | ACTACTGGACCGCACGATACC | CCGCATCACCTGGATACTGTTC |
| Epha3 | GCCTACCGCAAGTTCACATCAG | CTGTTCGCAAGTGACGCTGTAG |
| Epha4 | CGTCGGTGAACTTGGAATGGAG | AATGGATGATGGTGCTGCTTGG |
| PTEN | CCCACCACAGCTAGAACTTATC | CGTCCTTTCCCAGCTTTACA |
| GAPDH | TCACCATCTTCCAGGAGCGAGAC | TGAGCCCTTCCACAATGCCAAAG |

**Table S2.** All conditions used in animal experiment

| Name | SCI | Hydrogel | 3D printing | siRNA |
| --- | --- | --- | --- | --- |
| Intact | Non | Non | Non | Non |
| Ctrl | Yes | Non | Non | Non |
| GM-PEGDA | Yes | PEGDA | Non | Non |
| GM-RA4IV | Yes | RA4IV | Non | Non |
| siRNA@LNPs + GM-RA4IV | Yes | RA4IV | Non | Yes |
| siRNA@LNPs + GM-3Dpro | Yes | PEGDA&RA4IV | Yes | Yes |

**Supplementary References**

[1] M.J. Kwon, Y. Seo, H. Cho, H.S. Kim, Y.J. Oh, S. Genişcan, M. Kim, H.H. Park, E.-H. Joe, M.-H. Kwon, H.C. Kang, B.G. Kim, Nanogel-mediated delivery of oncomodulin secreted from regeneration-associated macrophages promotes sensory axon regeneration in the spinal cord, *Theranostics* **2022**, *12*, 5856.

[2] O. Avraham, R. Feng, E.E. Ewan, J. Rustenhoven, G. Zhao, V. Cavalli, Profiling sensory neuron microenvironment after peripheral and central axon injury reveals key pathways for neural repair, *eLife* **2021**, *0*, e68457.

[3] H. Wu, C. Xing, B. Yu, L. Guo, X. Dou, L. Gao, S. Yang, Y. Zhang, X. Gao, S. Li, B. Xia, T. Ma, Y. Hao, Y. Yang, X. Gao, Y. Wei, B. Xue, Q. Zhang, C. Feng, J. Huang, Metabolic reprogramming of neural stem cells by chiral nanofiber for spinal cord injury, *ACS Nano* **2025**, *19*, 4785.
